# Chromosome-level genome of black cutworm provides novel insights into polyphagy and seasonal migration in insects

**DOI:** 10.1186/s12915-022-01504-y

**Published:** 2023-01-05

**Authors:** Minghui Jin, Bo Liu, Weigang Zheng, Conghui Liu, Zhenxing Liu, Yuan He, Xiaokang Li, Chao Wu, Ping Wang, Kaiyu Liu, Shigang Wu, Hangwei Liu, Swapan Chakrabarty, Haibin Yuan, Kenneth Wilson, Kongming Wu, Wei Fan, Yutao Xiao

**Affiliations:** 1 Shenzhen Branch, Guangdong Laboratory of Lingnan Modern Agriculture, Key Laboratory of Gene Editing Technologies (Hainan), Ministry of Agriculture and Rural Affairs, Agricultural Genomics Institute at Shenzhen, Chinese Academy of Agricultural Sciences, Shenzhen, China; 2 The State Key Laboratory for Biology of Plant Diseases and Insect Pests, Institute of Plant Protection, Chinese Academy of Agricultural Sciences, Beijing, 100193 China; 3 College of Agronomy, Jilin Agricultural University, Changchun, 130118 China; 4 Department of Clinical Oncology, University of Hong Kong, Hong Kong (Special Administrative Region), Hongkong, 999077 China; 5 School of Life Sciences, Central China Normal University, Wuhan, 430079 China; 6 Lancaster Environment Centre, Lancaster University, Lancaster, LAI 4YQ UK

**Keywords:** Genome assembly, Comparative genomics, Transcriptome, Host adaptation, Migration, Cutworm

## Abstract

**Background:**

The black cutworm, *Agrotis ipsilon*, is a serious global underground pest. Its distinct phenotypic traits, especially its polyphagy and ability to migrate long distances, contribute to its widening distribution and increasing difficulty of control. However, knowledge about these traits is still limited.

**Results:**

We generated a high-quality chromosome-level assembly of *A. ipsilon* using PacBio and Hi-C technology with a contig N50 length of ~ 6.7 Mb. Comparative genomic and transcriptomic analyses showed that detoxification-associated gene families were highly expanded and induced after insects fed on specific host plants. Knockout of genes that encoded two induced ABC transporters using CRISPR/Cas9 significantly reduced larval growth rate, consistent with their contribution to host adaptation. A comparative transcriptomic analysis between tethered-flight moths and migrating moths showed expression changes in the circadian rhythm gene *AiCry2* involved in sensing photoperiod variations and may receipt magnetic fields accompanied by MagR and in genes that regulate the juvenile hormone pathway and energy metabolism, all involved in migration processes.

**Conclusions:**

This study provides valuable genomic resources for elucidating the mechanisms involved in moth migration and developing innovative control strategies.

**Supplementary Information:**

The online version contains supplementary material available at 10.1186/s12915-022-01504-y.

## Background

The globally distributed pest, black cutworm (BCW, Lepidoptera: Noctuidae), *Agrotis ipsilon*, is infamous for its omnivorousness and destructiveness [1–3]. Its detoxification enzyme system confers strong adaptability to toxic secondary metabolites in a wide range of host plants including cotton, wheat, maize, and other crops. The larvae cut off seedling stems at or below the growing point, leaving empty areas in the field (Additional file 2: Fig. S1) [2]. The larvae, which typically hide in litter or soil during the day and emerge at night, are difficult to control with pesticide sprays [4, 5].

Entomological radar and insect trapping studies have shown that *A.* *ipsilon* can migrate long distances [5–7], which enables insects to avoid harsh environments temporally and spatially and to cause large-scale outbreaks in suitable areas. Processes involved in the long-distance migration of diurnal butterflies have been studied. For example, the migration of the monarch butterfly, *Danaus plexippus*, involves a time-compensated sun compass for orientation and multiple physiological processes regulated by juvenile hormone (JH) [8–11]. However, unlike diurnal butterflies, little is known about genetic variations and molecular mechanisms that enable long-distance seasonal migration of nocturnal moths such as BCW.

Many species of Noctuidae, the largest family in Lepidoptera, are major agricultural pests worldwide. Genomes of some of these such as *Helicoverpa armigera* [12], *Spodoptera litura* [13], and *S.* *frugiperda* [14] have been sequenced and used to study insect adaptation and evolution, yet little genomic information is available for underground pests. Genomic and molecular data are needed to enable in-depth research on the mechanisms involved in migration and to develop management strategies for BCW.

Here, we describe the de novo assembly of a chromosome-level reference genome for *A.* *ipsilon* and identified gene families involved in adaptation to different host plants and studied their function. We also identified a suite of important genes that are involved in circadian clocks, JH regulation, and energy consumption, which are important for seasonal migration. Our results provide insights into the mechanisms that enable cutworm moth migration and essential information for designing safe, innovative control strategies.

## Results

### Chromosome-level genome assembly and annotation

**Fig. 1:**
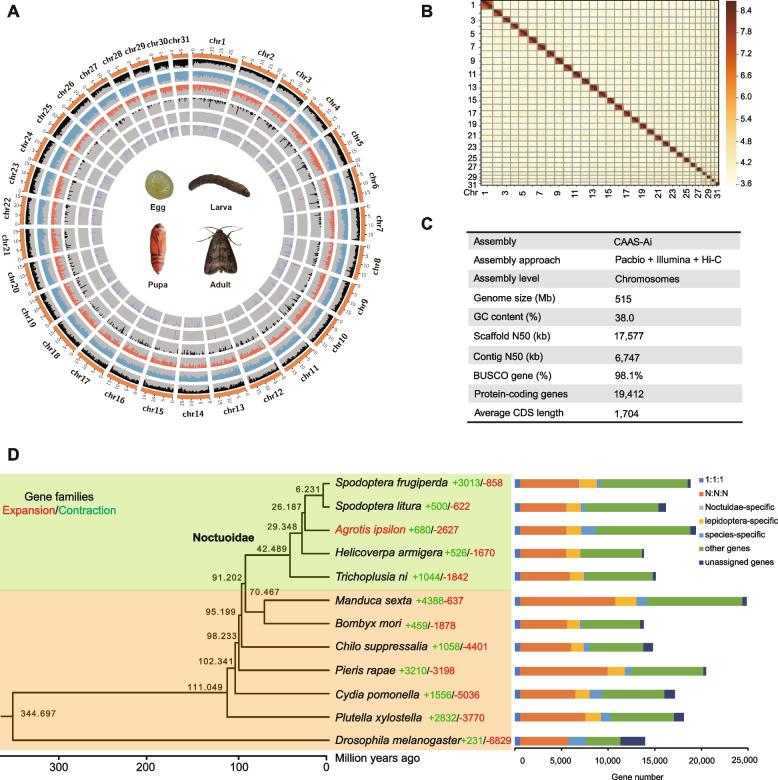
Chromosome-level genome assembly and evolutionary analysis of *Agrotis ipsilon*. **A** Integration of genomic and expression data for *A.* *ipsilon*. Track 1: 31 chromosomes of the *A.* *ipsilon* genome; track 2: distribution of guanine and cytosine (GC); track 3: distribution of transposon elements in chromosomes; track 4: distribution of coding genes; tracks 5–7: expression of organ-specific genes (from outside in head, pereon, and abdomen). **B** Genome-wide contact matrix from Hi-C data between each pair of the 31 chromosomes. **C** Major characteristics of the *A.* *ipsilon* genome. **D** Phylogenetic relationship and orthological comparison of 12 insects. Values on the nodes indicate the divergence time. The number of expanded gene families (red) and contracted gene families (green) are shown on the branches. Bars are subdivided to represent different types of orthologous groups. The ratio 1:1:1 indicates the common orthologs with a single copy in different species; N:N:N includes orthologous groups with different copy numbers in different species

We generated 49.4 Gb (96 ×) of Pacbio SMRT raw reads with an average read length of 15 kb, and 64 Gb (124 ×) of Illumina Hiseq paired-end reads using DNA extracted from a single 10-generation inbred male pupa of *A*. *ipsilon*. After quality filtering, the clean PacBio SMRT reads were then used to assemble the reference genomes with the long-read assembler Wtdbg2 [15]. The assembled genomes comprise 274 contigs with a contig N50 of 6.7 Mb (Additional file 1: Table S1). Estimated from the distribution of *k*-mer frequencies, the genome size of *A.* *ipsilon* was ~ 521 Mb (Additional file 2: Fig. S2). With the aid of Hi-C technology, 506.5 Mb (98.3%) sequences were anchored and arranged into 31 linkage groups, increasing the scaffold N50 to 17.58 Mb (Fig. 1A and B). The karyotyping analyses also showed that the chromosomes formed 31 discrete and separate bivalents (Additional file 2: Fig. S3). A final genome of 515 Mb was assembled with a contig N50 of 6.7 Mb and a scaffold N50 of 17.6 Mb (Fig. 1C). Our assembly has a higher degree of contiguity and completeness than previously published BCW assembly (scaffolds version) [16], i.e., contig N50 of 6.7 Mb vs 63 kb, representing an approximately 106-fold increase in average contig length (Additional file 1: Table S1).

We used the EVidence Modeler (EVM) pipeline to predict the protein-coding genes in the reference genome. In total, 19,412 gene models were predicted for the assembled genome of *A.* *ipsilon* (Fig. 1C). In an evaluation of completeness using Benchmarking Universal Single-Copy Orthologs (BUSCO), 98.1% of core genes from OrthoDB were identified as complete in the reference gene set (arthropoda v9), comparable to other published genomes [16]. Using functional databases, approximately 99.2% (19,256) of gene set in *A.* *ipsilon* were annotated (Additional file 2: Fig. S4).

### Genome evolution

To gain insights into an evolutionary perspective for *A.* *ipsilon*, we generated a phylogenetic tree by PhyML [17] based on 592 single-copy ortholog groups of 12 species (*Spodoptera frugiperda*, *Spodoptera litura*, *Agrotis ipsilon*, *Helicoverpa armigera*,* Trichoplusia ni*, *Manduca sexta*,* Bombyx mori*,* Chilo suppressalia*,* Pieris rapae*,* Cydia pomonella*,* Plutella xylostella*, and *Drosophila melanogaster*) and estimated divergence time using mcmctree (Fig. 1D). The results showed that the BCW have the last common ancestor with *D. melanogaster* ~ 345 million years ago (Mya), with *P.* *xylostella* ~ 111 Mya, with *B.* *mori* ~ 70 Mya, and with *H.* *arimigera* ~ 29 Mya (Fig. 1D).

To study the conservation of genomic structure, we identified interspecific syntenic genes in the *A.* *ipsilon* and *S.* *litura* using the MCScan pipeline (with parameter –w 5). We found a high syntenic relationship between *A.* *ipsilon* and *S.* *litura* (Additional file 2: Fig. S5). In addition, the amino acid identity of the pairs of syntenic genes between *A.* *ipsilon* and *S.* *litura* was 80.0%, showing high homology.

Orthologous genes were then investigated for adaptive evolution in the BCW. Utilizing pairwise protein sequence similarities, we conducted a gene family clustering analysis using orthoFinder [18]. We annotated 4920 N:N:N genes, 1517 Lepidoptera-specific genes, and 1592 species-specific genes (Fig. 1D).

The expansion and contraction of gene families are also thought to be important for adaptive phenotypic diversification. Compared with the common ancestor of *A.* *ipsilon* and *S.* *litura*, we identified 680 gene families that underwent expansion and 2627 that underwent contraction. KEGG analysis showed that the expanded genes were significantly enriched in metabolism pathways such as carbohydrate, amino acid, and lipid metabolism and transporter systems, and signal transduction (Additional file 2: Fig. S6A). Among the genes for signal transduction pathway, the FoxO family of transcription factors, which regulate the expression of genes in many important physiological events including cell cycle (CC), oxidative stress resistance/DNA repair (OR), immune-regulation (IR), and apoptosis (AP) [19], was chosen for a more detailed analysis of gene expansion (Additional file 2: Fig. S6B). Several key genes were found to have expanded in this pathway including those for insulin, phosphatidylinositol 3-kinase (p13K), and inhibitor of nuclear factor kappa-B kinase (IKKα/β) (Additional file 2: Fig. S6B). The expansion of signal transduction system genes might be associated with its defense against infection by microorganisms in the soil.

### Association of detoxification enzymes with host adaptations

**Fig. 2:**
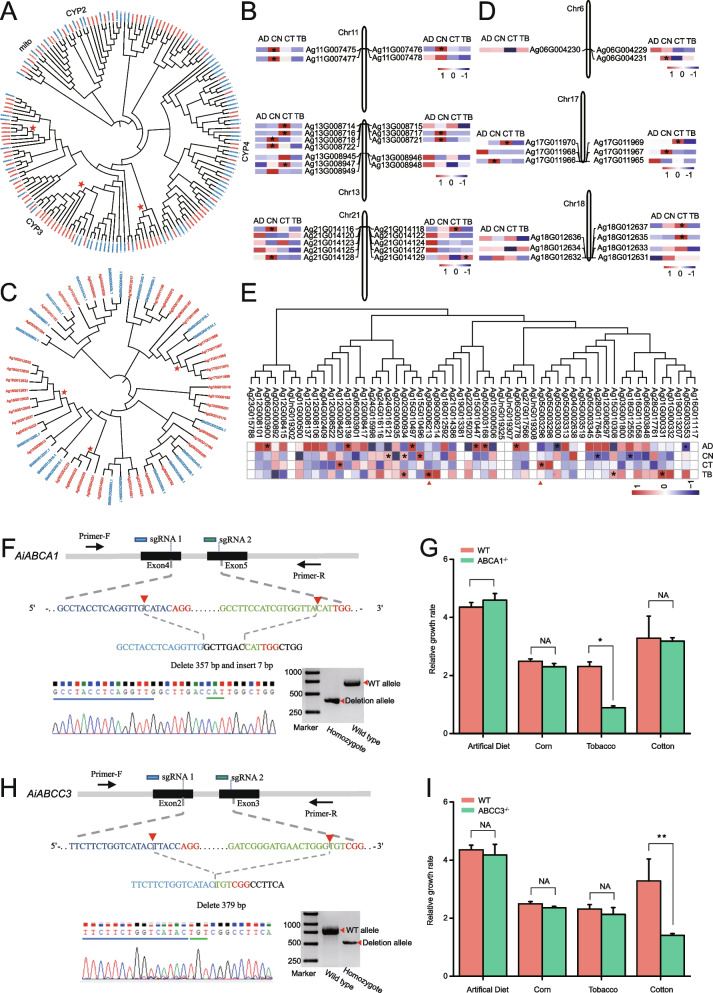
Detoxification genes related to host adaptation in *Agrotis ipsilon*. **A** Phylogenetic gene tree of P450s from *A.* *ipsilon* (red) and *Bombyx mori* (blue). Species-specific expanded clades in *A.* *ipsilon* are marked with red stars. **B** Expression profiles of expanded *A. ipsilon cytochrome P450* (*CYP*) gene clusters induced in larvae fed different diets. AD, artificial diet; CN, corn; CT, cotton; TB, tobacco (*|fold change|> 1.5, *p* < 0.05). **C** Phylogenetic tree of GSTs in *A. ipsilon* (red) and *B.* *mori* (blue). Species-specific expanded clades in *A.* *ipsilon* are marked with red stars. **D** Expression profiles of expanded *A. ipsilon* GST gene clusters induced in larvae fed different diets (*|fold change|> 1.5, *p* < 0.05). **E** Expression profiles of *A.* *ipsilon* ABC transporters induced in larvae fed different diets. AD, artificial diet; CN, corn; CT, cotton; TB, tobacco (*|fold change|> 1.5, *p* < 0.05). **F** Schematic diagram of sgRNA-targeted sites in *AiABCA1* gene. Exons are shown as black boxes; target site locations are marked by red triangles; PAM sequences are shown in red. Chromatograms show the nucleotide sequences for an *AiABCA1* homozygote. Agarose gel shows the PCR-based chromosomal deletion alleles of *AiABCA1*. **G** The relative growth rate of *ABCA1* mutants and wild type that fed on the different diets (means ± SE, *p < 0.05). **H** Schematic diagram of sgRNA-targeted sites in *AiABCC3* gene. Exons are shown as black boxes; target site locations are noted; PAM sequences are shown in red. Chromatograms show nucleotide sequences for an *AiABCC3* homozygote. Agarose gel of PCR-based chromosomal deletion alleles of *AiABCC3*. **I** The relative growth rate of *ABCC3* mutants and wild type that fed on different diets (means ± SE, **p < 0.01)

Agricultural pests, especially polyphagous insects, usually have an efficient detoxification system to overcome toxic plant defense chemicals [13]. This process usually involves multiple detoxification pathways, from detoxification of xenobiotics by enzymes [20] to enhanced excretion activity by ATP-binding cassette (ABC) transporters [21]. Here, we annotated 106 P450 genes in the *A.* *ipsilon* genome, among which P450 clans 3 and 4 showed large expansions compared with that in a model lepidopteran insect, the silkworm (Fig. 2A). Expression profile analysis of genes in major tissues and developmental stages showed that 74.5% (70/94) of the P450 genes were widely expressed across tissues and developmental stages (Additional file 2: Fig. S7). A distribution analysis showed at least 13 P450 clusters in the BCW genome (Additional file 2: Fig. S8). The largest P450 cluster (*CYP6AE*) was located on Chr21 and consisted of 10 *CYP* genes (Fig. 2A), which have been reported to be involved in detoxifying plant toxins and insecticides [22]. The transcriptomic analysis after larvae were allowed to feed on corn, cotton, and tobacco showed that three expanded P450 gene clusters were significantly induced (Fig. 2B), suggesting a link between the expansion of P450 gene clusters and increased tolerance to plant toxins [13, 23].

Moreover, 42 glutathione *S*-transferase (GST) genes were identified in the *A.* *ipsilon* genome, almost twice as many as in the *B.* *mori* genome. The GST superfamily comprises detoxification enzymes. The phylogenetic tree showed that several GST gene clusters were significantly expanded in the *A.* *ipsilon* genome compared with that of *B.* *mori* (Fig. 2C). Similar to the expression profiles of P450s, GST genes were also widely expressed in all developmental stages and various tissues (Additional file 2: Fig. S9). Distribution analysis showed 4 GST clusters in the BCW genome (Additional file 2: Fig. S10). The largest GST cluster was located on Chr18 and consisted of 7 GST genes (Fig. 2C). The expression of three expanded GST clusters was also significantly induced after larvae were allowed to feed on leaves from different host plants (Fig. 2D), indicating that the expansion of GSTs also contributed to host adaptation of this pest.

In addition to detoxifying enzymes that act directly on xenobiotics, ABC transporters are also involved in detoxification through the efflux of the xenobiotics, but their role in detoxification has been far less studied than that of detoxification enzymes. We annotated 56 ABC transporters in the *A.* *ipsilon* genome. Although the ABC gene family had not significantly expanded compared with the family in *B.* *mori* and other polyphagous pests, expression of the genes was highly induced after BCW had fed on corn, cotton, and tobacco (Fig. 2E). The expression patterns of each ABC transporter gene differed after feeding on the different hosts, and the expression of some ABC genes was host-specific. For example, *AiABCA1* (*Ag09G006213*) was highly induced in larvae that fed on tobacco (1.98-fold, *p* < 0.05), and *AiABCC3* (*Ag05G003296*) was highly induced in those that fed on cotton (2.05-fold, *p* < 0.01).

To further investigate the functions of ABCs, we used CRISPR/Cas9 genome editing to knockout *AiABCA1* and *AiABCC3*. Dual single-guide RNAs (sgRNAs) against *AiABCA1* and *AiABCC3* were designed. Individual freshly laid eggs were then injected with two in vitro transcribed sgRNA with 50 ng/μl of Cas9 protein. We then screened for homozygotes similar to our previous study [24]. After three generations of selection, we obtained stable homozygous *AiABCA1* and *AiABCC3* gene knockout strain ABCA1^−/−^ and ABCC3^−/−^, respectively (Fig. 2F, H). After CRISPR/Cas9-mediated ablation of *AiABCA1*, the relative growth rate of third instar larvae feeding on tobacco (which produces the secondary metabolite nicotine) of mutant strain ABCA1^−/−^ was significantly lower than that of the wild-type strain (Fig. 2G). Moreover, the growth rate of *AiABCC3* knockout strain ABCC3^−/−^ was significantly lower after feeding on cotton (which produces the secondary metabolite gossypol) compared with the wild-type strain (Fig. 2I). These results provide evidence that ABC transporters contribute to host adaptation.

### Analysis of seasonal long-distance migration of A. ipsilon

To investigate the molecular mechanism of long-distance migration of this pest using a comparative transcriptome analysis between migratory (wild-capture) and non-migratory moths (lab-rearing), we focused on three pathways essential for migration: circadian rhythms, JH regulation, and energy metabolism [9, 11].

### Circadian rhythms

**Fig. 3:**
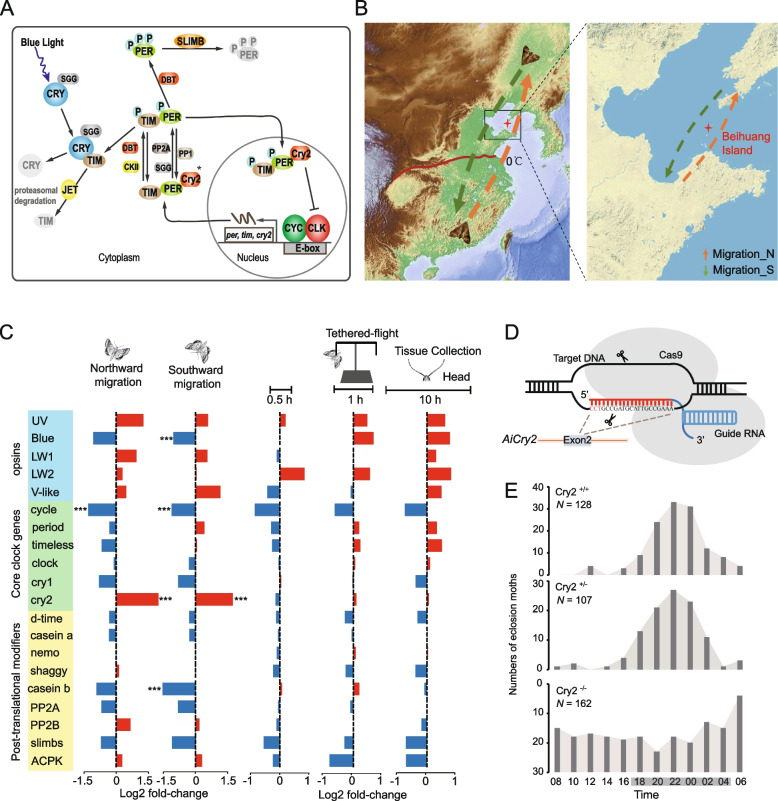
Circadian rhythm pathway involved in seasonal migration of *Agrotis ipsilon*. **A** Diagram of the proposed clock mechanism in *A.* *ipsilon*, including the core transcriptional feedback loop and the posttranscriptional regulation loop. Modified from Zhan et al. [9]. Heterodimers of clock (clk) and cycle (cyc) drive the transcription of period (per), timeless (tim), and mammalian-type cryptochrome (cry2). After 24 h, the PER, TIM, and CRY2 complexes cycle back into the nucleus, where CRY2 inhibits clk and cyc-mediated transcription. Casein kinase II (ckII), phosphatase 2A (pp2a), and doubletime (dbt) are involved in the posttranslational modifications of PER and TIM. Type-1 cryptochrome (cry1) mediates light entrainment and promotes TIM degradation. ∗ cry2 not present in all insects. B Location of Beihuang Island. Migrating moths of the black cutworm (BCW) were captured by vertical-pointing searchlight traps on the island during northward (from northern China to northeastern China; Migration_N) and southward migrations (from northeastern China to northern China; Migration_S). Red dotted line: northern boundary for overwintering of BCW. BCW cannot survive winters in northern China where temperatures fall below 0 ℃ (33 N). C Changes in expression of circadian rhythm genes in northward- and southward-migrating moths and tethered-flight moths compared with control strain. ***p < 0.001. D Diagram of A. ipsilon cry2 sgRNA-targeted site. sgRNA sequences are highlighted in red. E AiCry2 deficiency disrupts adult eclosion in constant darkness. Data for adult eclosion of wild type (Cry2+/+) and heterozygous (Cry2+/−) and homozygous (Cry2−/−) mutants were recorded every 2 h. Effect of genotype on eclosion time, one-way ANOVA: *p* < 0.004; Tukey’s post hoc test: Cry2+/+ vs. Cry2+/−, *p* > 0.05; Cry2+/+ vs. Cry2−/−, *p* < 0.05; Cry2+/− vs. Cry2−/−, *p* < 0.05. Black horizontal bars: subjective night; gray horizontal bars: subjective day

Circadian rhythms play an essential role in insect oviposition, endocrine activity, sexual behavior, development, and migration and have increased the evolutionary success of insects in various niches [25–27]. Firstly, we manually annotated genes related to the circadian clock based on orthologous genes in *D. melanogaster* and *D.* *plexippus*. We thus identified genes involved in the core transcriptional feedback loop, which includes *period* (*per*), *timeless* (*tim*), type-1 *cryptochrome* (*cry1*), *clock* (*clk*), and *cycle* (*cyc*) (Fig. 3A; Additional file 1: Table S2); genes encoding orthologs of the major proteins involved in the posttranscriptional regulation loop; and the important output regulator pigment-dispersing factor (PDF) (Additional file 1: Table S2). The results indicated that the circadian rhythm pathway was conserved in nocturnal and diurnal leipidopterans, although their behavior varies greatly.

We then asked whether the regulation of the circadian rhythm pathway changes in relation to seasonal migration. To investigate the potential involvement of circadian rhythms in BCW seasonal migration, we compared the transcriptomes in brain tissues of northward-migrating BCW moths vs non-migrating moths and of southward-migrating BCW moths vs non-migrating moths. The island where the migrating moths were captured is located in the center of Bohai Strait, an ideal collection site for long-distance insect migration [28] (Fig. 3B). After quantitative analysis of gene expression, we found diverse expression patterns in components of the circadian rhythm pathway (Fig. 3C). Before analyzing the core clock genes, we analyzed the phototransduction gene opsins. Among five identified opsin transcripts, those for UV, LW1, and V-like opsin were slightly but non-significantly upregulated in both northward- and southward-migrating moths, whereas the gene for blue opsin was downregulated in northward- and southward-migrating moths. Among the core clock genes, the *cycle* gene, which acts as a positive element to activate the transcription factors for *per*, *tim*, and *cry2*, was significantly downregulated in both northward- and southward-migrating moths. The *cry2* gene, which encodes the major transcriptional repressor of the clockwork feedback loop, was significantly upregulated in both northward- and southward-migrating moths. Moreover, the expression of casein b was downregulated in migrating moths (Fig. 3C).

To eliminate the impact of flight activities on circadian rhythms, we performed another comparative transcriptome analysis for brain tissues from moths after different durations tethered to a flight mill (Fig. 3C). Unlike migratory moths, the expression levels of blue opsin increased as the duration of the tethered flight increased. The expression levels of the *cycle* gene were downregulated similar to those in the migrating moths. Most interestingly, the expression of the *cry2* gene did not change in tethered-flight moths. The inducers for insect migration are complex, including seasonally variable daylengths, temperature, or food sources. Although tethered flight can simulate the flight state of insects, it cannot completely simulate the natural migration state because environmental changes that trigger migration may be perceived during the larval stage. So, we speculate that the downregulation of the *cycle* gene in migrating and tethered-flight moths may be related to the flying itself, whereas the upregulated *cry2* gene in migrating moths and lack of change in *cry2* in tethered-flight moths may be related to the absence of a trigger(s) for seasonal migration such as diurnal changes.

To verify the function of *cry2* genes, we knocked out the gene using the CRISPR/Cas9 system (Fig. 3D). After screening for homozygotes, we obtained a knockout strain (cry2^−/−^) with a stable homozygous cry2^−/−^ gene. To assess the influence of the knockout on circadian behavior, we examined the timing of pupal eclosion, which is a robust, tractable behavior under the control of the circadian clock [29, 30]. The results showed that the circadian timing of adult eclosion was significantly disrupted in strain cry2^−/−^, in comparison to that of the wild type (cry2^+/+^) and heterozygote type (cry2^+/−^) (*p* < 0.004) (Fig. 3E), suggesting that *cry2* has an important role(s) in the circadian clock of these insects and may be involved in the perception of diurnal changes that trigger seasonal migration.

The expression of *cry1*, a homologous gene of *cry2*, which encodes a protein that generally acts as a blue light receptor to fine-tune clock rhythm, was also analyzed in migrating moth and tethered-flight moths. Although the expression changes did not reach a significant level, it was downregulated in migrating moths and in tethered-flight moths after longer durations. This interesting phenomenon still needs further explanation. In addition, the expression level of the important output regulator PDF showed no significantly changed in migrating and tethered-flight moths (Additional file 2: Fig. S11).

Nocturnal migrants are reported to use the Earth’s magnetic field to locate destinations [31], but the magnetoreceptor (*MagR*) and *cry2* genes are presently the only genes known so far to encode proteins associated with magnetic perception [32, 33]. We thus characterized the ortholog of *MagR* in the BCW genome and found its expression was significantly upregulated in northward- (3.5-fold) and southward- (4.8-fold) migrating moths, but did not change in tethered-flight moths. The high expression of *cry2* and *MagR* genes in migrating moths may play important roles for perceiving magnetic fields.

### Juvenile hormone regulation

**Fig. 4:**
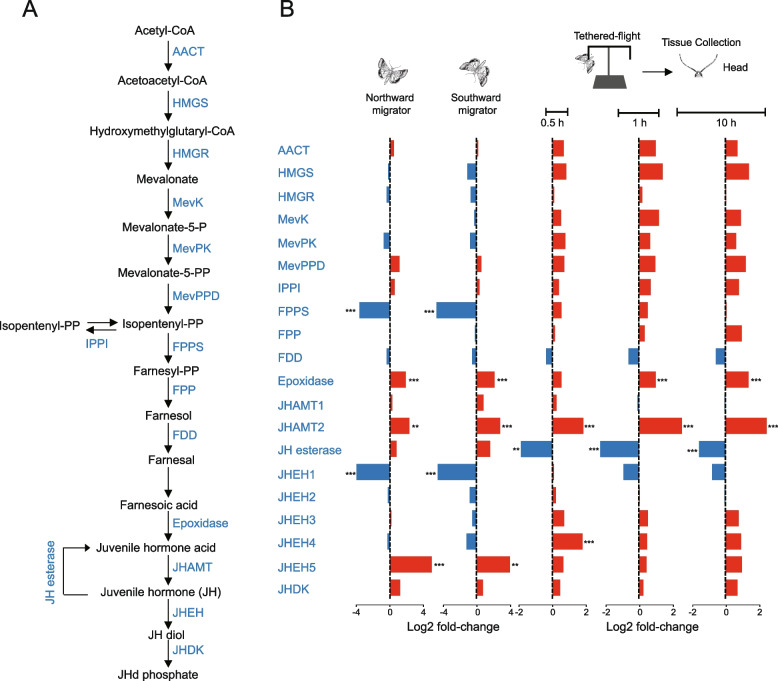
Juvenile hormone regulatory pathways.** A** Biosynthetic and degradation pathways for juvenile hormone (JH) in BCW genome. Enzymes are marked in blue. AACT, acetoacetyl-CoA thiolase; HMGS, HMG-CoA synthase; HMGR, HMG-CoA reductase; MevK, mevalonate kinase; MevPK, phosphomevalonate kinase; MevPPD, diphosphomevalonate decarboxylase; IPPI, IPP isomerase, FPPS, farnesyl PP synthase; FPP, farnesyl PP pyrophosphatase; FDD, farnesol dehydrogenase, JHAMT, *O*-methyltransferase; JHEH, JH epoxide hydrolase; JHDK, JH diol kinase. **B** Changes in expression of JH pathway genes in northward- and southward-migrating moths and in moths after tethered flights of different durations. **p < 0.01; ***p < 0.001

We then focused on genes involved in juvenile hormone (JH) biosynthesis because JH regulation is crucial for insects to coordinate reproductive and migratory physiological processes [34]. All the genes known to be involved in JH biosynthesis in insects [35] were identified in the BCW genome, and JH epoxide hydrolase (*JHEH*), which is involved in JH degradation, was expanded (Fig. 4A, Additional file 1: Table S3). We then compared the expression profiles of these genes among the northward- and southward-migrating moths and lab-reared, tethered-flight moths (Fig. 4B). The results showed that the genes in the northward and the southward migrators had similar expression patterns. Specifically, the expression of *FPPS* and *JHEH1* were significantly downregulated and *Epoxidase*, *JHAMT2* (key genes in JH synthesis), and *JHEH5* (involved in JH degradation) were significantly upregulated. Because the JH titer is maintained through the dynamic regulation of JH biosynthesis and degradation [34], we speculated that the migrating moths might have evolved a mechanism to maintain JH levels through the coordinate regulation of JH biosynthetic and degradation genes. In addition, we also analyzed changes in JH biosynthetic genes in moths over time during tethered flight (Fig. 4B). We found that the expression of 12 genes were upregulated (3 genes were significant) compared with resting-state moths, confirming that the JH pathway plays important roles in flight activity. Interestingly, the expression patterns of *Epoxidase* and *JHAMT2* genes were significantly upregulated in tethered-flight moths, similar to their expression in the migrating moths. These results indicated that these two genes may play important role in flight-related processes (e.g., for flight muscle development, energy metabolism regulation). The JH esterase gene, which belonged to another pathway of JH degradation, was significantly downregulated in tethered-flight moths. The JHEH1 gene was slightly downregulated (non-significantly) in tethered-flight moths, whereas other JHEHs were slightly upregulated (non-significantly). Apparently, the degradation rate of JH is slowed in both migrating moths and tethered-flight moths through the regulation of the expression of different key genes. Moreover, we identified two important JH signaling pathway genes, *Broad* and *Kr-h1*, and analyzed their expression pattern in migrating and tethered-flight moths. The results showed that the expression level of *Broad* was low in the head and thorax/abdomen and was not significantly changed in the migrating and the tethered-flight moths (Additional file 2: Fig. S12). *Kr-h1* was highly expressed in the head and thorax/abdomen and was significantly downregulated in migrating moths (Additional file 2: Fig. S12). In addition, considering these results and that the same genes for JH synthesis are upregulated in migrating moths and tethered-flight moths, we speculated that short flights and long-distance flights may have some similarities in JH regulation, but still have some differences, as insects evolved different strategies to adapt to different environments.

### Energy metabolism

**Fig. 5:**
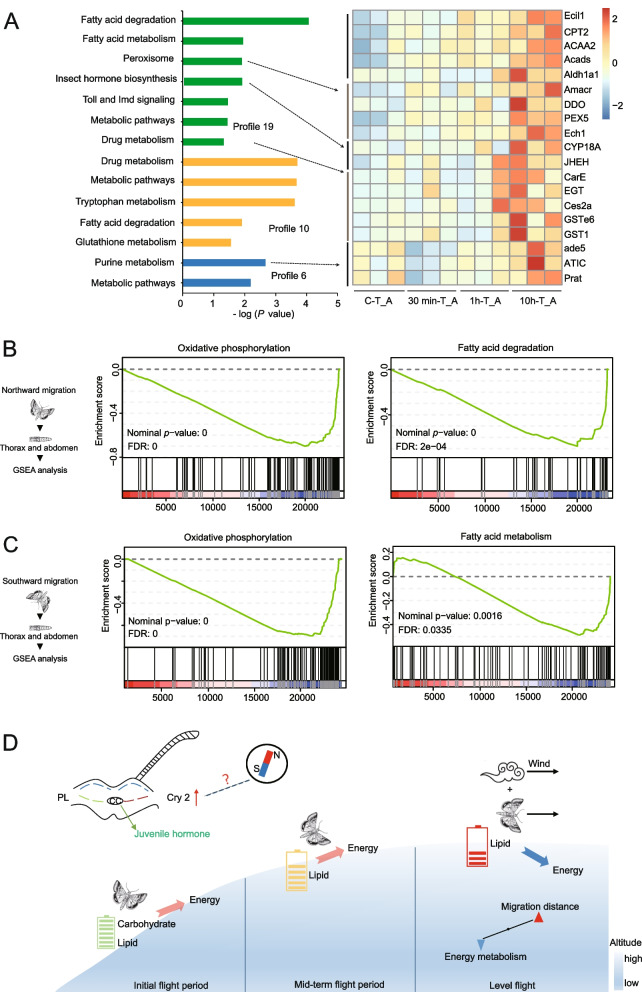
Transcriptome analysis of genes related to energy consumption during *Agrotis ipsilon *short-duration tethered flights and long-distance migratory flights.** A** KEGG pathway annotation of differentially expressed genes in profiles 19, 10, and 6. The *x*-axis represents the enrichment score. The heat map shows changes in the expression of candidate genes significantly enriched in an important pathway. **B** GSEA analysis of northward-migrating moths shows significant enrichment for oxidative phosphorylation and fatty acid degradation pathways; these pathways were downregulated, and FDR *q*-values are shown. **C** GSEA analysis of southward-migrating moths shows significant enrichment in oxidative phosphorylation and fatty acid metabolism pathways; these pathways were downregulated, and FDR *q*-values are shown. **D** The proposed model for high energy supply in *A.* *ipsilon* during flight. In the initial flight period, carbohydrate and lipid are the main sources of energy. In the mid-term flight period, carbohydrates are gradually depleted and lipid becomes the main source of energy. As the flight distance increases, the rate of energy metabolism decreases, and monsoon winds may help carry the moths to extend the flight time [40, 41]. The regulation of energy metabolism might be associated with the circadian rhythm and JH pathway

Energy metabolism obviously is critical for the long-distance seasonal migration of insects [11]. To characterize gross changes in energy metabolism during migration, we analyzed the transcriptome from the thorax/abdomen tissues of tethered-flight moths after short flights (Additional file 2: Fig. S13A) and from migrating moths (after a long flight). Firstly, we identified differentially expressed genes (DEGs) in the comparison of tethered-flight moths and non-flight moths and used them in a functional enrichment analysis in the program GSEA (Additional file 2: Fig. S14). After 30 min of flight, we analyzed the top 10 significantly enriched pathways based on FDR values (Additional file 1: Table S4) and found energy metabolism-related pathway glycolysis gluconeogenesis, carbon metabolism, oxidative phosphorylation, and fatty acid degradation pathway were significantly enriched, indicating that carbohydrates and lipids were used as a fuel source for the short flight (Additional file 2: Fig. S14). After longer flights (1 h and 10 h), the energy metabolism-related pathways were significantly enriched in genes for fatty acid degradation, fat digestion and absorption, and oxidative phosphorylation rather than carbohydrate metabolism (Additional file 2: Fig. S14). Thus, the moths seem likely to use lipids during long-distance flights, similar to *Locusta migratoria* [11]. In a trend analysis using four flight times, we identified four profiles that were significantly clustered (*p* < 0.01, Additional file 2: Fig. S13B, S15). Profile 19 contained 64 genes that had an increasingly higher expression as the flight times increased. Functional enrichment analysis showed a significant enrichment in genes for lipid metabolism, hormone regulation, peroxisome, and immune pathways (Fig. 5A). Profile 10 contained 55 genes that were only upregulated after 10 h of flight and also significantly enriched in lipid metabolism. The timing of the enrichment in these genes indicates that the moths regulate the expression of corresponding metabolic genes based on the type of energy substances to provide energy for long-distance flight. Profile 6 and profile 19 were respectively significantly enriched in purine and peroxisome metabolism pathways, which are involved in protection against reactive oxygen species generated by flight activity [36]. Among these significantly enriched pathways, we selected five pathways for specific gene expression analysis to identify potentially important genes involved in energy metabolism (Fig. 5A). In the heatmap of gene expression, these genes differed significantly between non-flight and flight stages, e.g., *Ecil1*, *CPT2*, *Acads*, and other genes in the fatty acid degradation pathway; *CYP18A* and *JHEH* genes in the insect hormone biosynthesis pathway; and *ATIC* and *Prat* genes in purine metabolism pathway. As flight duration increased, the expression level of these genes gradually increased.

To investigate metabolic pathways that are active in migrating moths, we analyzed the transcriptome in thorax/abdomen tissues from northward- and southward-migrating moths and non-migrating moths. The gene set enrichment analysis (GSEA) showed that pathways related to energy metabolism, including oxidative phosphorylation, lipid metabolism, glycolysis/gluconeogenesis, and citrate cycle were significantly downregulated in both northward- and southward-migrating moths (Fig. 5B, C, Additional file 2: Fig. S16). Similarly, flight metabolism is linked to dispersal ability in butterfly species [37, 38], and flight metabolic rates are lower in migratory populations than in non-migratory [39]. The captured migrating BCW moths had been flying for a long time, and part of their energy reserves had already been consumed. So, on the basis of the results from the moths after short-time flights (tethered-flight) and long-distance flights (migrating), we speculate that the migrating moths regulate their energy consumption by adjusting the expression of genes in energy metabolism pathways as their flight duration increases (Fig. 5D).

## Discussion

Although third-generation sequencing technologies have been widely used to generate long reads and numerous insect genomes have been published, few underground insect pests that are seasonal migrators and polyphagous have been similarly studied. Here, we used PacBio and Hi-C data to develop a 515-Mb chromosome-scale reference genome of *A.* *ipsilon*. Compared with a previous black cutworm genome assembly [16], with a contig N50 of 63 kb, our genome assembly with a contig N50 length of ~ 6.75 Mb was greatly improved. This BCW genome will pave the way for in-depth biological studies of BCW and help to develop effective strategies to control the pest.

Detoxification of plant secondary metabolites is crucial for the ecological adaptation of polyphagous pests to varied host plants. We identified the main detoxification gene families and investigated the expression of these genes after the larvae fed on different host plants. We found that the CYPs and GST genes, which introduce or release functional groups, thereby increasing the reactivity and hydrophilicity of toxins, were greatly expanded in BCW and significantly induced by the different hosts. Although genes in the ABC gene family, which facilitate the active transport of toxins across membranes for excretion, had not significantly expanded, expression of these genes was also highly induced by different hosts. The expression of ABC transporter genes in other insects is similarly induced by different host plants [42, 43]. To further investigate the function of ABC transporters, we knocked out either *AiABCA1* or *AiABCC3* in BCW using the CRISPR/Cas9 system. Interestingly, the bioassay results showed that the larval growth rate of knockout strain ABCA1 was significantly slower compared to the wild-type strain after they fed on tobacco, whereas the growth of knockout strain ABCC3 was significantly slower after it fed on cotton. We speculated that knocking out an ABC transporter gene makes the mutant strain defective in transporting xenobiotics (e.g., nicotine in tobacco, gossypol in cotton) and larval growth slows. These detoxification enzymes might be valuable targets for BCW management.

Unraveling the molecular mechanisms that enable the seasonal migration of insects is a challenging but fascinating task [9, 10, 44]. Seasonal migration of insects may be regulated by a complex network, including sensing the seasonal variation of habitats such as decreasing day length and cooler temperatures, which appear to trigger the migration, balancing life span, reproduction, and other physiological activities; detecting flight direction during migration; adjusting energy metabolism during migration; and so on. In this study, we identified genes involved in the circadian rhythm pathway and found that *AiCry2* was specifically upregulated in migrating moths (both in northward- and southward-migrating moths). Knockout of *AiCry2* using the CRISPR/Cas9 system significantly influenced eclosion, which has a circadian behavior. Moreover, the expression of *MagR*, which is associated with magnetic perception, was also upregulated in migrating moths. So, we speculate that the clock gene *AiCry2* is needed for migration to sense seasonal diurnal variations or magnetic fields in cooperation with MagR [32, 33]. Among the genes involved in JH regulation, JH biosynthesis genes *Expoxidase* and *JHATM2* were upregulated in both migrating moths and tethered-flight moths, indicating that JH is important for flight activities. For genes involved in energy metabolism, the results showed that carbohydrates were used as the main fuel source during short flights (as simulated with tethered-flight moths), whereas lipids were the fuel source during long flights (migrating moths). In addition, energy metabolism rates in migrating moths decreased, perhaps due to the depletion of the stored energy sources as the light time increased. Because insect migration is often coincident with monsoon winds, insects flying at certain altitudes can use the monsoon winds to assist their flight and conserve energy [40, 41, 45]. We speculate that the migrating moths adjust energy consumption by regulating energy metabolism pathways according to flight duration (Fig. 5D).

## Conclusions

Using our high-quality chromosome-level genome assembly for BCW, we compared genomes among different insect species and transcriptomes among different host diets and different flight durations, providing evidence on gene expansions that enabled this polyphagous insect to become a deleterious pest and adjust to soil environments and seasonal changing conditions. The complex molecular regulation of seasonal migration of BCW involves circadian rhythms, JH biosynthesis, and energy metabolism. This better understanding of the genetic basis of host adaptability and seasonal migration provides abundant target genes associated with specific biological processes and will facilitate the development of effective strategies to control *A.* *ipsilon*.

## Methods

### Insects and genomic sequencing

An inbred strain of *A*. *ipsilon*, collected in Taian city (Shandong Province, China) in 2012, was developed by successive single-pair sib mating for 10 generations and reared on an artificial diet in a controlled insect chamber (25 ± 2 ℃, 75 ± 10% RH, 14L:10D).

A single male pupa was selected to extract genomic DNA using a Genomic DNA kit (Qiagen) for both PacBio sequencing (PacBio SMRT) and Illumina sequencing (Illumina HiSeq 2500). The quality of the DNA was checked using a NanoDrop One UV–Vis spectrophotometer (Thermo Fisher Scientific) and Qubit 3.0 Fluorometer (Invitrogen), and high-integrity DNA molecules were measured using 1% agarose gel electrophoresis. For PacBio sequencing, a library with ~ 20-kb insert was constructed using PacBio SMRT and sequenced using the PacBio Sequel system (Pacific Biosciences). For Illumina sequencing, a short paired-end DNA library with a 350-bp insert was constructed using standard Illumina protocols, then sequenced.

### Genome assembly

We filtered the Illumina raw reads by trimming the adapter sequence and low-quality parts using in-house software clean_adapter and clean_lowqual, resulting in clean and high-quality reads with an average error rate of < 0.001. For the PacBio raw data, the short subreads (< 5 kb) were filtered out, and only one representative subread was retained for each PacBio read. The clean PacBio reads were used to assemble the reference genome with wtdbg2 (version 2.3) [15]. Then, we used Arrow (version 2.2.2) with default parameters to polish the reference genome. In addition, the Illumina reads were aligned with the contigs by BWA-MEM [46], and single base errors in the reference contigs were corrected using Pilon v1.21 [47] with parameters -fx bases, -nonpf, -minqual 20. We used the arthropoda gene set (odb9) to assess the completeness of the genome assembly by Benchmarking Universal Single-Copy Ortholog (BUSCO).

### Chromosome karyotype analysis

The testes were excised from 5th instar larvae 4 h after they had been injected with 0.03% (w/v) colchicine. The testes were soaked in H_2_O for 2 h, then fixed in Cano’s fixed liquid for 24 h, washed twice in PBS, and stored in 75% (v/v) ethanol until use. For observations, the organs were washed twice with H_2_O, placed on a glass slide, and then stained with Giemsa solution for 20–30 min. The samples were observed and photographed using a Nikon microscope (Tokyo, Japan).

### Hi-C sequencing

To finally ligate the scaffolds to chromosomes, Hi-C technology was used to capture the chromosome conformations using a fifth instar larva. The Hi-C library was prepared using a previously published protocol and sequenced using the Illumina HiSeq 2500 platform. After quality control, clean Hi-C reads were first mapped to the contig assembly by Bowtie2 (version 2.2.3) [48], then filtered using the default setting in HIC-PRO (version 2.7.8) [49]. LACHESIS [50] was used for clustering, ordering, and orienting the contigs into scaffolds. To access the accuracy of the scaled-up genome assembly, we cut the chromosomes predicted by LACHESIS into bins of equal length (100 kb) and constructed a heatmap based on the interaction signals revealed by valid mapped read pairs between bins, which was then visualized using HIC-PRO (version 3.1).

### Transcriptome library preparation and sequencing for genome annotation

The RNeasy mini kit (Qiagen, Shanghai, China) was used to isolate total RNA. RNA quality and quantity of each sample were determined using a NanoPhotometer spectrophotometer (Implen, CA, USA) and Qubit RNA Assay Kit in a Qubit 2.0 Fluorometer (Life Technologies, CA, USA). The samples include eight tissues (epicuticle, head, hemolymph, fat body, Malpighian tube, hindgut, midgut, foregut) from fifth instar larvae and eight developmental stages (egg, 1st instar larva, 3rd instar larva, 4th instar larva, 5th instar larva, pupa, male adult, and female adult). Raw sequencing data were generated using the Illumina HiSeq 2500 platform with the paired-end 150 (PE150) strategy.

### Genome annotation

A de novo repeat library for the genome was constructed using RepeatModeler v. 1.0.4. Transposable elements (TEs) in the cutworm genome were identified by RepeatMasker v4.0.6 using both the Repbase library and the de novo library. Tandem repeats in the genome were identified using Tandem Repeats Finder v4.07b. For gene prediction for the genome, we integrated evidence from ab initio predictions, homology-based searches, and RNA sequencing (RNA-seq) using EVidence Modeler v1.1.1 [51]. De novo prediction of coding genes was performed using AUGUSTUS with hint files. For homology-based prediction, over 20,000 amino acid sequences for Noctuidae were downloaded from NCBI and aligned to the genome using exonerate v2.2.0. For RNA-seq, the clean data were mapped to the reference genome using TOPHAT v2.1.0 and the gene regions were extracted with CUFFLINKS v2.2.1. Subsequently, these three types of results were integrated using EvidencModeler. Then, these gene models were annotated using RNA-seq data and the UniProt and NR databases. Gene models were then retained if they had at least one piece of supporting evidence from the UniProt database, NR database, and RNA-seq data. We annotated each genome for gene functions by aligning the protein sequences with sequences in the National Center for Biotechnology Information (NCBI) NR, UniProt, COG, and KEGG databases using BLASTp v2.3.0 + and an *E*-value cutoff of 10^−5^ and choosing the best hits. The KEGG database was used for pathway analysis and functional classification, and InterProScan [52] was used to assign preliminary GO terms and IPR domains to the gene models for each genome.

### Evolution analysis

Duplicated genomic fragments were identified using MCscanX [53], requiring at least five paralogous gene pairs per collinear block, and the duplicate_gene_classifier in MCscanX was implemented to classify the origins of the duplicate genes into different types. Orthologous and paralogous gene families were assigned using 12 insect species (*Spodoptera frugiperda*, *Spodoptera litura*, *Agrotis ipsilon*, *Helicoverpa armigera*,* Trichoplusia ni*,* Manduca sexta*,* Bombyx mori*,* Chilo suppressalia*,* Pieris rapae*, *Cydia pomonella*, *Plutella xylostella*, and *Drosophila melanogaster*) and OrthoFinder [18] with default parameters.

Gene families that contained only one gene for each species were selected to construct the phylogenetic tree. The protein sequences of each gene family were independently aligned using MUSCLE v3.8.31 [54] and then concatenated into one super-sequence. The phylogenetic tree was constructed by maximum likelihood (ML) using PhyML v3.0 [17]. The Bayesian-relaxed molecular clock approach was adopted to estimate the neutral evolutionary rate and species divergence time using MCMCTree in PAML v4.9 [55].

### Whole-genome synteny

Whole-genome synteny between *A.* *ipsilon* and *Spodoptera litura* was estimated. Amino acid sequences of the proteins were aligned using BLASTP (v2.6.0) (*E*-value < 1e^−10^); then, MCScanX was used with default parameters to construct chromosome collinearity blocks between species.

### Gene family expansion and contraction

The constructed phylogenetic tree with predicted divergence time and the identified families was analyzed using CAFE v.4.2 to perform gene family expansion/contraction analysis. We used *p* < 0.05 as the criterion for a significant change in the gene families. A KEGG enrichment analysis was conducted using OmicShare tools.

### Gene family analysis

To identify members of the P450, GST, and ABC transporter gene families, we first downloaded reference protein sequences of lepidopteran P450s, GSTs, and ABC transporters from NCBI GenBank. Then, BLASTP v2.6.0 (*E*-value < 1E^−5^) was used to find candidate sequences in the *A.* *ipsilon* genome assembly. We also confirmed the presence of candidate sequences against sequences from the Pfam database (*E*-value < 1E^−5^) using HMMER [56] v.3.2.1. The protein sequences were aligned using MUSCLE, and the phylogenetic trees were constructed using MEGA X with the neighbor-joining method.

### Effect of host plant diets on transcriptomes

Third instar larvae were individually reared in 24-well plates for 2 days on leaves of corn, cotton, and tobacco or an artificial diet as a control, then frozen in liquid nitrogen, and stored at − 80℃ for RNA-seq. Twelve larvae were pooled as one biological replicate; three biological replicates were used for each treatment.

### Collection of migrating moths

Migrating individuals of BCW were collected on Beihuang Island at the Beihuang Experimental Station of the Chinese Academy of Agricultural Sciences, Shandong Province, China. This small, isolated island is in the center of Bohai Strait (38°24′ N; 120°55′ E) and on a migratory pathway of many insects migrating between the northeastern and southern, central, and northern regions of China [41]. Migrating BCW moths were captured using a vertical-pointing searchlight trap. Northward- and southward-migrating moths were captured in June and September 2020, respectively. The captured moths were frozen in liquid nitrogen and stored at − 80°C. During sample transfer, samples were stored in a Trizol reagent and transported with dry ice.

Lab-reared moths were used as the control of non-migratory moths. To ensure the reliability of the results, we took several measures: (1) adjusted the temperature and photoperiod in the incubator to resemble the field environment when rearing the lab strain, (2) evaluated the lab-reared moths in short and long tethered flights to eliminate errors caused by flight activity itself, (3) mainly focused on pathways that are related to migration, and (4) focused on the genes that differed significantly in expression in both northern and southern migration.

### Tethered flight

Flight experiments were conducted in September 2020 using the lab-reared population. In brief, the FXMD-24-USB flight mill was placed in an artificial climate chamber, and a single BCW adult was attached to an apparatus [57]. The climate chamber was then completely darkened, and moths were allowed to engage freely in tethered flight. The flight experiments were turned for 0 h (control), 0.5, 1, and 10 h; then, moths were frozen in liquid nitrogen and stored at − 80℃ until used for RNA-seq.

### RNA extraction and sequencing for analyzing the mechanism of migration

Heads of the migrating moths and tethered-flight moths were excised from the whole moth. The total RNA of the heads and the rest of the body were extracted using an RNeasy mini kit. Each treatment consisted of three biological samples (12 insects per treatment). Paired-end read 150-bp sequencing was performed using Illumina Novaseq 6000 platform by Gene Denovo Biotechnology Co. (Guangzhou China). After filtering by fastp, the clean reads were mapped to our reference genome using HISAT2.2.4 with “-rna-standness RF” and other parameters set as a default. FPKM value was calculated to quantify the expression abundance using RSEM software [58]. Differentially expressed genes (DEGs) (FDR < 0.05 and |log2 fold change |≥ 1.5) were identified using the edgeR package version 3.12.1 [59].

In order to perform a trend analysis, gene expression patterns were analyzed to cluster genes with similar expression patterns for multiple samples. For DEGs, the expression data for each sample (in the order of treatment) were normalized to 0, log2(v1/v0), log2(v2/v0), and then clustered using STEM software [60]. The program parameters were as follows: maximum unit change in model profiles between time points = 1, maximum output profiles number = 20, and minimum ratio of fold change of DEGs ≥ 2.0. The clustered profiles with *p* < 0.05 were considered to be significant profiles. Then, the DEGs in each profile were subjected to KEGG analysis.

Gene set enrichment analysis (GSEA) was performed using the software GSEA [61] to identify whether a set of genes in specific KEGG pathways shows significant differences in two groups. Briefly, we input gene expression matrices and ranked genes by the signal-to-noise normalization method. Then, enrichment scores and *p*-values were calculated using default parameters.

### Knockout of AiCry2, AiABCA1, and AiABCC3 gene using CRISPR/Cas9

The sgRNA target sequences were selected at exons of the *AiCry2*,* AiABCA1*, and *AiABCC3* gene using the sgRNAcas9 design tool (Additional file 1: Table S5), then checked in a search of the *A*. *ipsilon* genome and GenBank database: no potential off-target sites were identified. Template DNA was made using PCR-based fusion of two oligonucleotides with the T7 promoter. The PCR reaction was performed using a GeneArt Precision gRNA Synthesis Kit (Thermo Fisher Scientific, Shanghai, China), and the reaction mixture contained 12.5 μL of Phusion High-Fidelity PCR Master Mix (2 ×), 1 μL Tracr Fragment + T7 Primer Mix, 1 μL of 0.3 μmol/L Target F/R oligonucleotide mix, and 10.5 μL Nuclease-free water. PCR was performed at 98 °C 10 s, 32 cycles of 98 °C 10 s; 60 °C 30 s and 72 °C 15 s; and 72 °C 10 min. In vitro transcription was also performed with the kit mentioned above according to the manufacturer’s instructions. Cas9 protein (GeneArt Platinum Cas9 Nuclease) was purchased from Thermo Fisher Scientific.

Freshly laid eggs (within 1 h) were collected and injected individually as described previously [62] with about 1.5 nL of a mixture of sgRNA (150 ng/µL) and Cas9 protein (50 ng/µL) using a Nanoject III (Drummond, Broomall, PA, USA). Microinjection was completed in 1 h. Injected eggs were incubated at 25 °C and 65% RH for 2–3 days until they hatched.

Primers were designed flanking the CRISPR target sites (Additional file 1: Table S5) and used in a PCR with the genomic DNA of an individual insect. The PCR amplicons were sent to Sangon Biotech (Shanghai, China) for direct sequencing. When double peaks were observed around the cut site, PCR products were TA-cloned and sequenced to determine the exact mutation type.

### Bioassay

Eclosion was assessed as previously described with slight modifications [29] using larvae that were reared with 16 L:8D at 26 ± 1 °C with 60 ± 10% relative humidity through pupation; then, pupae were transferred to constant darkness. Eclosion behavior of the wild-type (Cry2^+/+^), heterozygous (Cry2^+/−^), and homozygous (Cry2^−/−^) mutants was recorded continuously over time using a camera with night vision. Eclosion times were analyzed for differences among genotypes with a Student’s *t*-test and one-way ANOVA using SPSS software (25.0, IBM, Armonk, NY, USA) and plotted as 2-h bins.

Feeding on different host plants: third instar larvae of the wild-type and knockout strain (ABCA1^−/−^ and ABCC3^−/−^) were weighed (M1) and fed different host plants for 2 days. Sixteen individuals were included in each treatment with three replications. Larvae were weighed again (M2) to calculate relative growth rate [(M2-M1)/M1]. Mean relative growth rates among diet treatments were compared for significant differences using Student’s *t*-test.

## Supplementary Information

**Additional file 1: Table S1. **Comparisons of our genomic assembly with previous published genomic assembly of *Agrostis ipsilon*. **Table S2.** Circadian clock components in genome of *Agrosis ipsilon*. **Table S3.** Juvenile hormone regulatory pathway in genome of *Agrosis ipsilon. ***Table S4.** Top 10 GSEA enriched pathways after different durations of tethered-flight moths of *Agrostis ipsilon*. **Table S5.** Primers used in this study.

**Additional file 2: Fig. S1.** Life cycle of *Agrotis ipsilon* and crop damage. **Fig. S2.** Distribution of 17-mer freque*ncy in Agrotis ipsilon. ***Fig. S3.** Chromosomes of *Agrotis ipsilon* in cell of testis from fifth instar larvae. Thirty-one pairs (2*n* = 62) were present at diakinesis during meiosis. **Fig. S4.** Venn plot of gene function in five databases in *Agrotis ipsilon*. **Fig. S5.** Dot plots of syntenic orthologous between *A. ipsilon* and *S. litura*. **Fig. S6.** KEGG analysis of rapidly expanded gene families in *Agrostis ipsilon*. **Fig. S7.** Expression profiles of P450 genes in different tissues and developmental stages of *Agrostis ipsilon*. **Fig. S8.** Distribution of P450 genes on the chromosomes of *A. ipsilon*. **Fig. S9.** Expression profiles of GST genes in different tissues and developmental stages of* Agrostis ipsilon*. **Fig. S10.** Distribution of GST genes on the chromosomes of *Agrostis ipsilon*. **Fig. S11.** The expression level of pigment-dispersing factor (PDF) in heads of migrating moths and tethered-flight moths. **Fig. S12.** The expression level of JH signaling pathway genes, *Broad* and *Kr-h1*, in different tissues of migrating moths and tethered-flight moths (*p < 0.05, **p < 0.01). **Fig. S13. **RNA-seq of tethered-flight. **Fig. S14. **GSEA after different durations of tethered-flight moths of *Agrostis ipsilon*. **Fig. S15.** Trend analysis of DEGs after different durations of tethered flight. **Fig. S16.** GSEA analysis of southward and northward migrating moths.

**Additional file 3. **Raw gel image is shown in Additional File 3.

## Data Availability

All raw sequence data generated during this study have been deposited at NCBI as a BioProject under accession PRJNA595758 of *Agrotis ipsilon*. The genome assembly and annotation files were uploaded to the NCBI (accession number: PRJNA905188) and available at the website ftp://ftp.agis.org.cn/Four.cutworms.genome/.The RNA-seq raw data have been deposited in the Sequence Read Archive (SRA) database with accession code PRJNA868426 (different development stages and tissues), PRJNA868049 (different host plants treatment), and PRJNA870230 (migration moths). The raw gel image is shown in Additional file 3.

## Abbreviations

BCW: Black cutworm
JH: Juvenile hormone
BUSCO: Benchmarking Universal Single-Cope Orthologs
TEs: Transposable elements
KEGG: Kyoto Encyclopedia of Genes and Genomes
CC: Cell cycle
OR: Oxidative stress resistance/DNA repair
AP: Apoptosis
p13K: Phosphatidylinositol 3-kinase
ABC: ATP-binding cassette

## References

[1] Liu YQ, Fu XW, Mao LM, Xing ZL, Wu KM (2016). Host plants identification for adult Agrotis ipsilon, a long-distance migratory insect. Int J Mol Sci.

[2] Nyamwasa I, Li K, Rutikanga A, Rukazambuga DNT, Zhang S, Yin J (2018). Soil insect crop pests and their integrated management in East Africa: a review. Crop Prot.

[3] Zhang J, Li H, Tan J, Wei P, Yu S, Liu R (2019). Transcriptome profiling analysis of the intoxication response in midgut tissue of Agrotis ipsilon larvae to Bacillus thuringiensis Vip3Aa protoxin. Pestic Biochem Physiol.

[4] Abdullah MAF, Moussa S, Taylor MD, Adang MJ (2009). Manduca sexta (Lepidoptera: Sphingidae) cadherin fragments function as synergists for Cry1A and Cry1C Bacillus thuringiensis toxins against noctuid moths Helicoverpa zea, Agrotis ipsilon and Spodoptera exigua. Pest Manag Sci.

[5] Zeng J, Liu YQ, Zhang HW, Liu J, Jiang YY, Wyckhuys KAG (2020). Global warming modifies long-distance migration of an agricultural insect pest. J Pest Sci.

[6] Liu YQ, Fu XW, Feng HQ, Liu ZF, Wu KM (2015). Trans-regional migration of Agrotis ipsilon (Lepidoptera: Noctuidae) in North-East Asia. Ann Entomol Soc Am.

[7] Feng HQ, Wu KM, Cheng DF, Guo YY (2003). Radar observations of the autumn migration of the beet armyworm Spodoptera exigua (Lepidoptera: Noctuidae) and other moths in northern China. B Entomol Res.

[8] Reppert SM, Guerra PA, Merlin C (2016). Neurobiology of monarch butterfly migration. Annu Rev Entomol.

[9] Zhan S, Merlin C, Boore JL, Reppert SM (2011). The monarch butterfly genome yields insights into long-distance migration. Cell.

[10] Reppert SM, de Roode JC (2018). Demystifying monarch butterfly migration. Curr Biol.

[11] Wang XH, Fang XD, Yang PC, Jiang XT, Jiang F, Zhao DJ (2014). The locust genome provides insight into swarm formation and long-distance flight. Nat Commun.

[12] Pearce SL, Clarke DF, East PD, Elfekih S, Gordon KHJ, Jermiin LS (2017). Genomic innovations, transcriptional plasticity and gene loss underlying the evolution and divergence of two highly polyphagous and invasive Helicoverpa pest species. BMC Biol.

[13] Cheng TC, Wu JQ, Wu YQ, Chilukuri RV, Huang LH, Yamamoto K (2017). Genomic adaptation to polyphagy and insecticides in a major East Asian noctuid pest. Nat Ecol Evol.

[14] Gui FR, Lan TM, Zhao Y, Guo W, Dong Y, Fang DM (2020). Genomic and transcriptomic analysis unveils population evolution and development of pesticide resistance in fall armyworm Spodoptera frugiperda. Protein Cell.

[15] Ruan J, Li H (2019). Fast and accurate long-read assembly with wtdbg2. Nat Methods.

[16] Wang YH, Fang GQ, Chen X, Cao YH, Wu NN, Cui Q (2021). The genome of the black cutworm Agrotis ipsilon. Insect Biochem Mol Biol.

[17] Guindon S, Dufayard JF, Lefort V, Anisimova M, Hordijk W, Gascuel O. New algorithms and methods to estimate maximum-likelihood phylogenies: assessing the performance of PhyML 3.0. Syst Biol. 2010;59:307–21. 10.1093/sysbio/syq010 20525638

[18] Emms DM, Kelly S (2015). OrthoFinder: solving fundamental biases in whole genome comparisons dramatically improves orthogroup inference accuracy. Genome Biol.

[19] Krafczyk N, Klotz LO (2022). FOXO transcription factors in antioxidant defense. IUBMB Life.

[20] Li XC, Schuler MA, Berenbaum MR (2007). Molecular mechanisms of metabolic resistance to synthetic and natural xenobiotics. Annu Rev Entomol.

[21] Dermauw W, Van Leeuwen T (2014). The ABC gene family in arthropods: comparative genomics and role in insecticide transport and resistance. Insect Biochem Mol Biol.

[22] Wang HD, Shi Y, Wang L, Liu S, Wu SW, Yang YH (2018). CYP6AE gene cluster knockout in Helicoverpa armigera reveals role in detoxification of phytochemicals and insecticides. Nat Commun.

[23] Jin M, Liao C, Fu X, Holdbrook R, Wu K, Xiao Y (2019). Adaptive regulation of detoxification enzymes in Helicoverpa armigera to different host plants. Insect Mol Biol.

[24] Jin MH, Yang YC, Shan YX, Chakrabarty S, Cheng Y, Soberon M, et al. Two ABC transporters are differentially involved in the toxicity of two Bacillus thuringiensis Cry1 toxins to the invasive crop pest Spodoptera frugiperda (J. E. Smith). Pest Manag Sci. 2021;77:1492–501. 10.1002/ps.6170 33145907

[25] Niepoth N, Ke G, de Roode JC, Groot AT (2018). Comparing behavior and clock gene expression between caterpillars, butterflies, and moths. J Biol Rhythm.

[26] Panda S, Hogenesch JB, Kay SA (2002). Circadian rhythms from flies to human. Nature.

[27] Yerushalmi S, Green RM (2009). Evidence for the adaptive significance of circadian rhythms. Ecol Lett.

[28] Chang H, Guo JL, Fu XW, Hou YM, Wu KM (2019). Orientation behavior and regulatory gene expression profiles in migratory Agrotis ipsilon (Lepidoptera: Noctuidae). J Insect Behav.

[29] Merlin C, Beaver LE, Taylor OR, Wolfe SA, Reppert SM (2013). Efficient targeted mutagenesis in the monarch butterfly using zinc-finger nucleases. Genome Res.

[30] Markert MJ, Zhang Y, Enuameh MS, Reppert SM, Wolfe SA, Merlin C. Genomic access to monarch migration using TALEN and CRISPR/Cas9-mediated targeted mutagenesis. G3-Genes Genom Genet. 2016;6:905–15. 10.1534/g3.116.027029 PMC4825660 26837953

[31] Guerra PA, Reppert SM (2015). Sensory basis of lepidopteran migration: focus on the monarch butterfly. Curr Opin Neurobiol.

[32] Qin S, Yin H, Yang C, Dou Y, Liu Z, Zhang P (2016). A magnetic protein biocompass. Nat Mater.

[33] Xu JJ, Wan GJ, Hu DB, He J, Chen FJ, Wang XH (2016). Molecular characterization, tissue and developmental expression profiles of cryptochrome genes in wing dimorphic brown planthoppers. Nilaparvata lugens Insect Sci.

[34] Xiao HJ, Fu XW, Liu YQ, Wu KM (2016). Synchronous vitellogenin expression and sexual maturation during migration are negatively correlated with juvenile hormone levels in Mythimna separata. Sci Rep.

[35] Belles X, Martin D, Piulachs MD (2005). The mevalonate pathway and the synthesis of juvenile hormone in insects. Annu Rev Entomol.

[36] Magwere T, Pamplona R, Miwa S, Martinez-Diaz P, Portero-Otin M, Brand MD (2006). Flight activity, mortality rates, and lipoxidative damage in Drosophila. J Gerontol a-Biol.

[37] Niitepold K, Smith AD, Osborne JL, Reynolds DR, Carreck NL, Martin AP (2009). Flight metabolic rate and Pgi genotype influence butterfly dispersal rate in the field. Ecology.

[38] Mitikka V, Hanski I (2010). Pgi genotype influences flight metabolism at the expanding range margin of the European map butterfly. Ann Zool Fenn.

[39] Zhan S, Zhang W, Niitepold K, Hsu J, Haeger JF, Zalucki MP (2014). The genetics of monarch butterfly migration and warning colouration. Nature.

[40] Drake VA, Farrow RA (1988). The influence of atmospheric structure and motions on insect migration. Annu Rev Entomol.

[41] Reynolds DR, Smith AD, Chapman JW (2008). A radar study of emigratory flight and layer formation by insects at dawn over southern Britain. B Entomol Res.

[42] Dermauw W, Osborne EJ, Clark RM, Grbic M, Tirry L, Van Leeuwen T (2013). A burst of ABC genes in the genome of the polyphagous spider mite Tetranychus urticae. BMC Genomics.

[43] Bretschneider A, Heckel DG, Vogel H (2016). Know your ABCs: Characterization and gene expression dynamics of ABC transporters in the polyphagous herbivore Helicoverpa armigera. Insect Biochem Mol Biol.

[44] Gu ZR, Pan SK, Lin ZZ, Hu L, Dai XY, Chang J (2021). Climate-driven flyway changes and memory-based long-distance migration. Nature.

[45] Feng HQ, Wu KM, Ni YX, Cheng DF, Guo YY (2006). Nocturnal migration of dragonflies over the Bohai Sea in northern China. Ecol Entomol.

[46] Li H, Durbin R (2009). Fast and accurate short read alignment with Burrows-Wheeler transform. Bioinformatics.

[47] Walker BJ, Abeel T, Shea T, Priest M, Abouelliel A, Sakthikumar S (2014). Pilon: an integrated tool for comprehensive microbial variant detection and genome assembly improvement. PLoS ONE.

[48] Langmead B, Salzberg SL (2012). Fast gapped-read alignment with Bowtie 2. Nat Methods.

[49] Servant N, Varoquaux N, Lajoie BR, Viara E, Chen CJ, Vert JP (2015). HiC-Pro: an optimized and flexible pipeline for Hi-C data processing. Genome Biol.

[50] Burton JN, Adey A, Patwardhan RP, Qiu RL, Kitzman JO, Shendure J (2013). Chromosome-scale scaffolding of de novo genome assemblies based on chromatin interactions. Nat Biotechnol.

[51] Haas BJ, Salzberg SL, Zhu W, Pertea M, Allen JE, Orvis J (2008). Automated eukaryotic gene structure annotation using EVidenceModeler and the program to assemble spliced alignments. Genome Biol.

[52] Quevillon E, Silventoinen V, Pillai S, Harte N, Mulder N, Apweiler R (2005). InterProScan: protein domains identifier. Nucleic Acids Res.

[53] Tang HB, Bowers JE, Wang XY, Ming R, Alam M, Paterson AH (2008). Perspective - synteny and collinearity in plant genomes. Science.

[54] Edgar RC (2004). MUSCLE: multiple sequence alignment with high accuracy and high throughput. Nucleic Acids Res.

[55] Yang ZH (2007). PAML 4: phylogenetic analysis by maximum likelihood. Mol Biol Evol.

[56] Potter SC, Luciani A, Eddy SR, Park Y, Lopez R, Finn RD (2018). HMMER web server: 2018 update. Nucleic Acids Res.

[57] Ge SS, He LM, He W, Yan R, Wyckhuys KAG, Wu KM (2021). Laboratory-based flight performance of the fall armyworm. Spodoptera frugiperda J Integr Agr.

[58] Li B, Dewey CN (2011). RSEM: accurate transcript quantification from RNA-Seq data with or without a reference genome. BMC Bioinformatics.

[59] Robinson MD, McCarthy DJ, Smyth GK (2010). edgeR: a Bioconductor package for differential expression analysis of digital gene expression data. Bioinformatics.

[60] Ernst J, Bar-Joseph Z (2006). STEM: a tool for the analysis of short time series gene expression data. BMC Bioinformatics.

[61] Subramanian A, Tamayo P, Mootha VK, Mukherjee S, Ebert BL, Gillette MA (2005). Gene set enrichment analysis: a knowledge-based approach for interpreting genome-wide expression profiles. Proc Natl Acad Sci USA.

[62] Jin MH, Xiao YT, Cheng Y, Hu J, Xue CB, Wu KM (2019). Chromosomal deletions mediated by CRISPR/Cas9 in Helicoverpa armigera. Insect Sci.

