# Supplementary material for: Chromosome-level genome of black cutworm provides novel insights into polyphagy and seasonal migration in insects
Source: BMC Biol. 2023 Jan 5;21:2. doi: 10.1186/s12915-022-01504-y (PMC9814246; doi:10.1186/s12915-022-01504-y)
Supplement: Supplementary file 1 — Additional file 1: Table S1. Comparisons of our genomic assembly with previous published genomic assembly of Agrostis ipsilon. Table S2. Circadian clock components in genome of Agrosis ipsilon. Table S3. Juvenile hormone regulatory pathway in genome of Agrosis ipsilon. Table S4. Top 10 GSEA enriched pathways after different durations of tethered-flight moths of Agrostis ipsilon. Table S5. Primers used in this study. [file 12915_2022_1504_MOESM1_ESM.docx]

**Supplementary Materials**

**Chromosome-level genome of black cutworm provides novel insights into polyphagy and seasonal migration in insects**

Minghui Jin^1,2#^, Bo Liu^1#^, Weigang Zheng^1,3#^, Chonghui Liu^1,6#^, Zhenxing Liu^1#^, He Yuan^1,2#^, Xiaokang Li^2^, Chao Wu^1^, Ping Wang^1^, Kaiyu, Liu^4^, Shigang Wu^1^, Hangwei Liu^1^, Swapan Chakrabarty^1^, Haibin Yuan^3^, Kenneth Wilson^5^, Kongming Wu^2*^, Wei Fan^1*^, Yutao Xiao^1*^

1 Shenzhen Branch, Guangdong Laboratory of Lingnan Modern Agriculture, Agricultural Genomics Institute at Shenzhen, Chinese Academy of Agricultural Sciences, Shenzhen 518120, China

2 The State Key Laboratory for Biology of Plant Diseases and Insect Pests, Institute of Plant Protection, Chinese Academy of Agricultural Sciences, Beijing 100193, China

3 College of Agronomy, Jilin Agricultural University, Changchun 130118, China

4 School of Life Sciences, Central China Normal University, Wuhan 430079, China

5 Lancaster Environment Centre, Lancaster University, Lancaster, LAI 4YQ, United Kingdom.

6 Department of Clinical Oncology, University of Hong Kong, Hong Kong (Special Administrative Region), Hongkong999077, China

^#^These authors contributed equally to this work.

^*^Correspondence: Yutao Xiao ([xiaoyutao@caas.cn](mailto:xiaoyutao@caas.cn)), Wei Fan ([fanwei@caas.cn](mailto:fanwei@caas.cn)), Kongming Wu ([wukongming@caas.cn](mailto:wukongming@caas.cn)).

**Supplementary Tables**

**Table S1. Comparisons of our genomic assembly with previous published genomic assembly of *Agrostis ipsilon***

|  | CAAS-Ai | ASM419385v1(Wang, et al. 2021) |
| --- | --- | --- |
| Assembly size (Mb) | 515 | 500 |
| Assembly method | Pacbio+Illumina+Hi-C | Illumina |
| Assembly level | Chromosomes | Scaffolds |
| Number of Scaffolds | 31 | 7971 |
| Scaffold N50 (Mb) | 17.6 | 1.0 |
| Number of Contigs | 274 | 36,270 |
| Contig N50 (Mb) | 6.7 | 0.06 |
| Protein-coding | 19,412 | 14,801 |
| GC (%) | 38.0 | 37.6 |
| BUSCOs (%) | 98.1 | 97.9 |

**Table S2. Circadian clock components in genome of *Agrosis ipsilon*.**

| Gene symbol | *Agrostis ipsion* | *Danaus plexippus* | *Drosophila melanogaster* |
| --- | --- | --- | --- |
| ***Core feedback loop clock genes*** | | | |
| clock | Ag01G000330 | DPGLEAN10878 | NP_523964 |
| cycle | Ag01G000013 | DPGLEAN21608 | NP_524168 |
| period | Ag01G000292 | DPGLEAN18581 | NP_525056 |
| timeless | Ag10G006894 | DPGLEAN08466 | NP_722912 |
| cryptochrome-1 | Ag09G006450 | DPGLEAN06594 | NP_732407 |
| cryptochrome-2 | Ag05G003308 | DPGLEAN05008 |  |
| ***Post-translational modifiers*** | | | |
| double-time | Ag09G006200 | DPGLEAN17544 | NP_524602 |
|  | Ag24G016412 |  |  |
| nemo | Ag15G010011 | DPGLEAN22338 | NP_729318 |
| shaggy | Ag03G001874 | DPGLEAN11950 | NP_476716 |
| casein kinase_IIa | Ag02G001178 | DPGLEAN04623 | NP_524918 |
| caseinkinase_IIb | Ag14G009752 | DPGLEAN17707 | NP_001014731 |
| protein phospohatase 2A-B’ | Ag20G013538 | DPGLEAN18342 | NP_732296 |
|  | Ag20G013722 |  |  |
| supernumerary limbs | Ag31G018975 | DPGLEAN12824 | NP_524430 |
| jetlag | Ag17G011392 | DPGLEAN15995 | NP_001036332 |
| AMP-activated protein kinase | Ag21G014209 | DPGLEAN08679 | NP_996327 |
| ***Output*** |  |  |  |
| Pigment dispersing factor | AG15G010296 | DP0GS205081 |  |

**Table S3. Juvenile hormone regulatory pathway in genome of *Agrosis ipsilon*.**

| Gene ID | symbol |
| --- | --- |
| Ag14G009580 | AACT |
| Ag18G012480 | HMGS |
| Ag29G018304 | HMGR |
| Ag21G014373 | MevK |
| Ag13G008951 | MevPK |
| Ag17G011515 | MevPPD |
| Ag20G013772 | IPPI |
| Ag18G012389 | FPPS2 |
| Ag18G012387 | FPP |
| Ag21G014208 | FDD |
| Ag04G002527 | Epoxidase |
| Ag12G008402 | JHAMT1 |
| Ag12G008403 | JHAMT2 |
| Ag25G016896 | JH esterase |
| Ag25G016497 | JHEH1 |
| Ag25G016496 | JHEH2 |
| Ag25G016495 | JHEH3 |
| Ag25G016494 | JHEH4 |
| Ag25G016493 | JHEH5 |
| Ag08G005608 | JHDK |
| Ag10G007075 | Kr-h1 |
| Ag16G011285 | Broad-complex |

**Table S4. Top 10 GSEA enriched pathway after different durations of tethered-flight moths of *Agrostis ipsilon*.**

|  | name | SIZE | ES | FDR q-val |
| --- | --- | --- | --- | --- |
| 30min | Glycolysis / Gluconeogenesis | 63 | 0.7232124 | 0 |
|  | Pyruvate metabolism | 57 | 0.69568413 | 0 |
|  | Carbon metabolism | 158 | 0.7496493 | 0 |
|  | Drug metabolism - cytochrome P450 | 90 | 0.6222502 | 1.45E-04 |
|  | Oxidative phosphorylation | 113 | 0.57564694 | 6.44E-04 |
|  | Fatty acid degradation | 45 | 0.6427933 | 0.002857849 |
|  | Porphyrin and chlorophyll metabolism | 69 | 0.5894689 | 0.003889385 |
|  | Glycerolipid metabolism | 73 | 0.58497435 | 0.004577393 |
|  | Steroid hormone biosynthesis | 56 | 0.5964326 | 0.007196986 |
|  | Insect hormone biosynthesis | 33 | 0.5090443 | 0.008316285 |
|  | name | SIZE | ES | FDR q-val |
| 1h | Ribosome | 111 | 0.6758372 | 0 |
|  | Oxidative phosphorylation | 113 | 0.52797896 | 3.02E-04 |
|  | Glycerolipid metabolism | 73 | 0.5926579 | 4.53E-04 |
|  | Fat digestion and absorption | 33 | 0.6568965 | 6.63E-04 |
|  | Parkinson | 114 | 0.51386315 | 0.001113717 |
|  | Fatty acid degradation | 45 | 0.5850871 | 0.00576823 |
|  | Steroid biosynthesis | 28 | 0.6062618 | 0.015406847 |
|  | Legionellosis | 36 | 0.56719023 | 0.016703948 |
|  | Glycerophospholipid metabolism | 67 | 0.48585296 | 0.028351715 |
|  | Valine, leucine and isoleucine degradation | 46 | 0.51067615 | 0.037862103 |
|  | name | SIZE | ES | FDR q-val |
| 10h | Ribosome | 111 | 0.6463948 | 0 |
|  | Valine, leucine and isoleucine degradation | 46 | 0.6515985 | 0 |
|  | Fatty acid degradation | 45 | 0.61840755 | 6.20E-04 |
|  | cGMP - PKG signaling pathway | 100 | -0.5598869 | 0.005068569 |
|  | Oxidative phosphorylation | 113 | 0.48133254 | 0.006194992 |
|  | Oxytocin signaling pathway | 90 | -0.540859 | 0.009071269 |
|  | ECM-receptor interaction | 31 | -0.6321704 | 0.009571364 |
|  | Gastric acid secretion | 41 | -0.59931797 | 0.01048054 |
|  | Vascular smooth muscle contraction | 81 | -0.5342067 | 0.012132783 |
|  | Fat digestion and absorption | 49 | 0.53427315 | 0.012549002 |

**Table S5. Primers used in this study.**

| Primer name | Sequence (5’ to 3’) | Purpose |
| --- | --- | --- |
| ABCA1-sgRNA1 | GCCTACCTCAGGTTGCATAC | sgRNAs |
| ABCA1-sgRNA2 | GCCTTCCATCGTGGTTACAT |  |
| ABCC3-sgRNA1 | TTCTTCTGGTCATACTTACC |  |
| ABCC3-sgRNA2 | GATCGGGATGAACTGGGTGT |  |
| CRY2-sgRNA | GCCGATGCATTGCCGAAA |  |
| A1-F | ACCAGAAACTTGCTGCTAGT | detect mutation |
| A1-R | CGCGTACCCCTACTTTCCCA |  |
| C3-F | TATGGTTTGGTTTGGCAGCA |  |
| C3-R | TATTGGCAACCCGTCTCCT |  |
| CRY2-F | CAGCGACGTTCAGATGTG |  |
| CRY2-R | TTGTGGTCTCGCACGCGGC |  |
