# Supplementary material for: Chromosome-level genome of black cutworm provides novel insights into polyphagy and seasonal migration in insects
Source: BMC Biol. 2023 Jan 5;21:2. doi: 10.1186/s12915-022-01504-y (PMC9814246; doi:10.1186/s12915-022-01504-y)
Supplement: Supplementary file 2 — Additional file 2: Fig. S1. Life cycle of Agrotis ipsilon and crop damage. Fig. S2. Distribution of 17-mer frequency in Agrotis ipsilon. Fig. S3. Chromosomes of Agrotis ipsilon in cell of testis from fifth instar larvae. Thirty-one pairs (2n = 62) were present at diakinesis during meiosis. Fig. S4. Venn plot of gene function in five databases in Agrotis ipsilon. Fig. S5. Dot plots of syntenic orthologous between A. ipsilon and S. litura. Fig. S6. KEGG analysis of rapidly expanded gene families in Agrostis ipsilon. Fig. S7. Expression profiles of P450 genes in different tissues and developmental stages of Agrostis ipsilon. Fig. S8. Distribution of P450 genes on the chromosomes of A. ipsilon. Fig. S9. Expression profiles of GST genes in different tissues and developmental stages of Agrostis ipsilon. Fig. S10. Distribution of GST genes on the chromosomes of Agrostis ipsilon. Fig. S11. The expression level of pigment-dispersing factor (PDF) in heads of migrating moths and tethered-flight moths. Fig. S12. The expression level of JH signaling pathway genes, Broad and Kr-h1, in different tissues of migrating moths and tethered-flight moths (*p < 0.05, **p < 0.01). Fig. S13. RNA-seq of tethered-flight. Fig. S14. GSEA after different durations of tethered-flight moths of Agrostis ipsilon. Fig. S15. Trend analysis of DEGs after different durations of tethered flight. Fig. S16. GSEA analysis of southward and northward migrating moths. [file 12915_2022_1504_MOESM2_ESM.docx]

**Supplementary Materials**

**Chromosome-level genome of black cutworm provides novel insights into polyphagy and seasonal migration in insects**

Minghui Jin^1,2#^, Bo Liu^1#^, Weigang Zheng^1,3#^, Chonghui Liu^1,6#^, Zhenxing Liu^1#^, He Yuan^1,2#^, Xiaokang Li^2^, Chao Wu^1^, Ping Wang^1^, Kaiyu, Liu^4^, Shigang Wu^1^, Hangwei Liu^1^, Swapan Chakrabarty^1^, Haibin Yuan^3^, Kenneth Wilson^5^, Kongming Wu^2*^, Wei Fan^1*^, Yutao Xiao^1*^

1 Shenzhen Branch, Guangdong Laboratory of Lingnan Modern Agriculture, Agricultural Genomics Institute at Shenzhen, Chinese Academy of Agricultural Sciences, Shenzhen 518120, China

2 The State Key Laboratory for Biology of Plant Diseases and Insect Pests, Institute of Plant Protection, Chinese Academy of Agricultural Sciences, Beijing 100193, China

3 College of Agronomy, Jilin Agricultural University, Changchun 130118, China

4 School of Life Sciences, Central China Normal University, Wuhan 430079, China

5 Lancaster Environment Centre, Lancaster University, Lancaster, LAI 4YQ, United Kingdom.

6 Department of Clinical Oncology, University of Hong Kong, Hong Kong (Special Administrative Region), Hongkong999077, China

^#^These authors contributed equally to this work.

^*^Correspondence: Yutao Xiao ([xiaoyutao@caas.cn](mailto:xiaoyutao@caas.cn)), Wei Fan ([fanwei@caas.cn](mailto:fanwei@caas.cn)), Kongming Wu ([wukongming@caas.cn](mailto:wukongming@caas.cn)).

**Supplementary Figures**


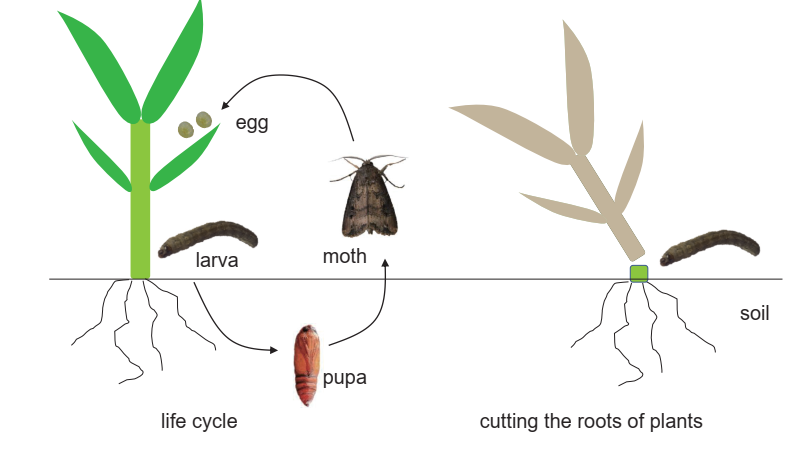


**Supplementary Figure S1. Life cycle of *Agrotis ipsilon* and crop damage.**

**Supplementary Figure S2. Distribution of 17-mer freque*ncy in Agrotis ipsilon.*** We used Illumina reads from the short-insert libraries to calculate the kmer frequencies. The 17-kmer distribution showed a major peak. Based on the number of kmers and relative kmer depth, we predicted that the genome size of *Agrotis ipsilon* is 521 Mb using the formula: Genome size= Kmer_Number/Peak_Depth.


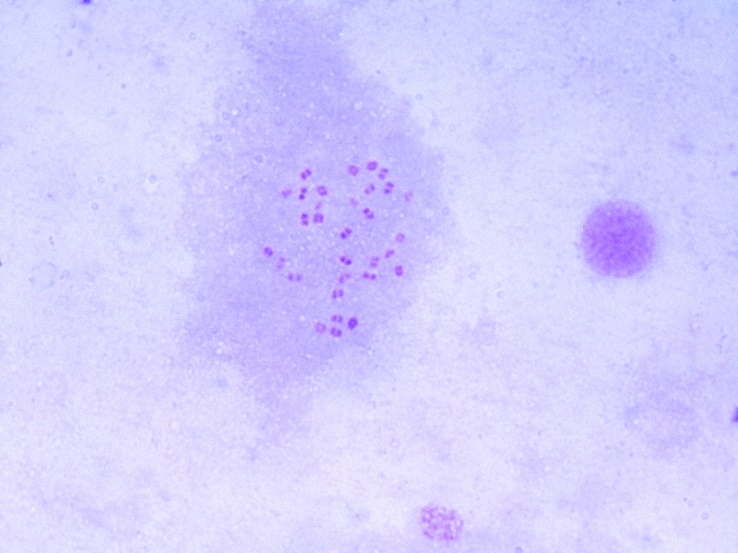


**Supplementary Figure S3.** **Chromosomes of *Agrotis ipsilon* in cell of testis from fifth instar larvae.** Thirty-one pairs (2*n* = 62) were present at diakinesis during meiosis.


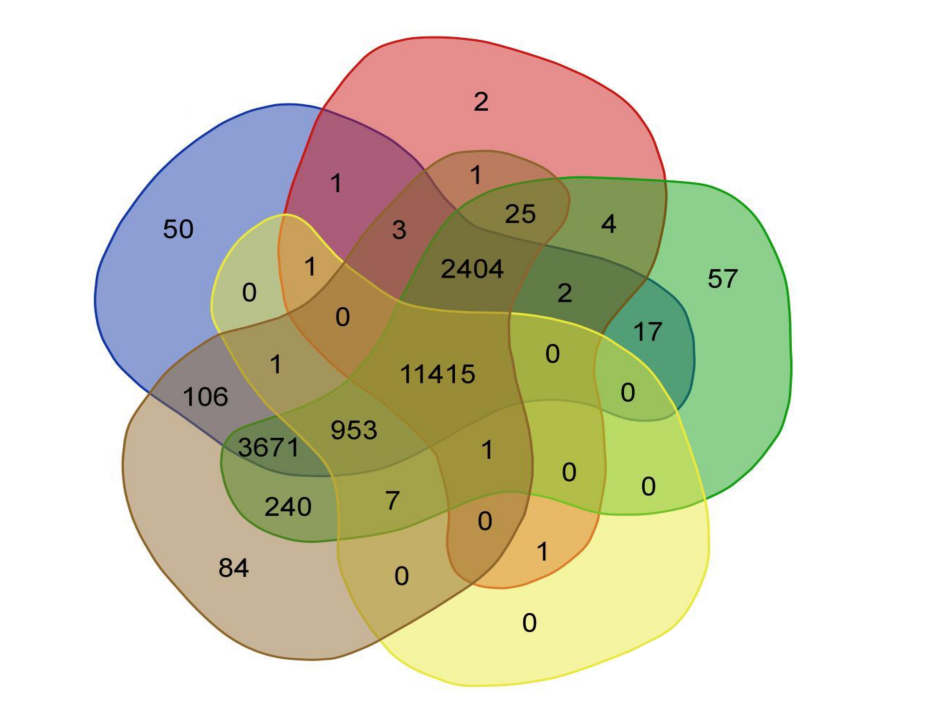


**Supplementary Figure S4. Venn plot of gene function in five databases in *Agrotis ipsilon*. The five databases including KEGG, eggNOG, NR, Uniprot, and COG databases.**


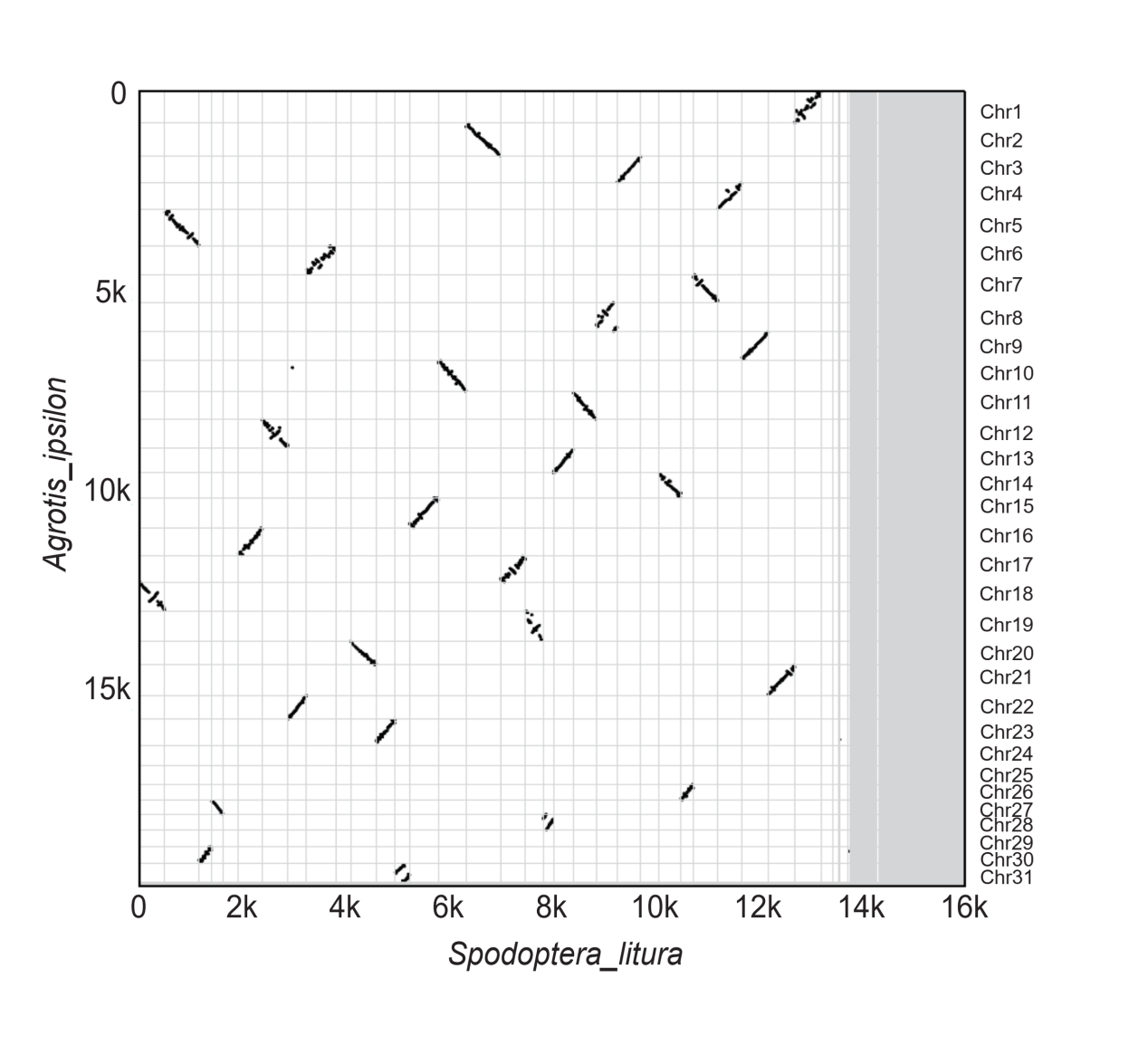


**Supplementary Figure S5. Dot plots of syntenic orthologous between *A. ipsilon* and *S. litura.***


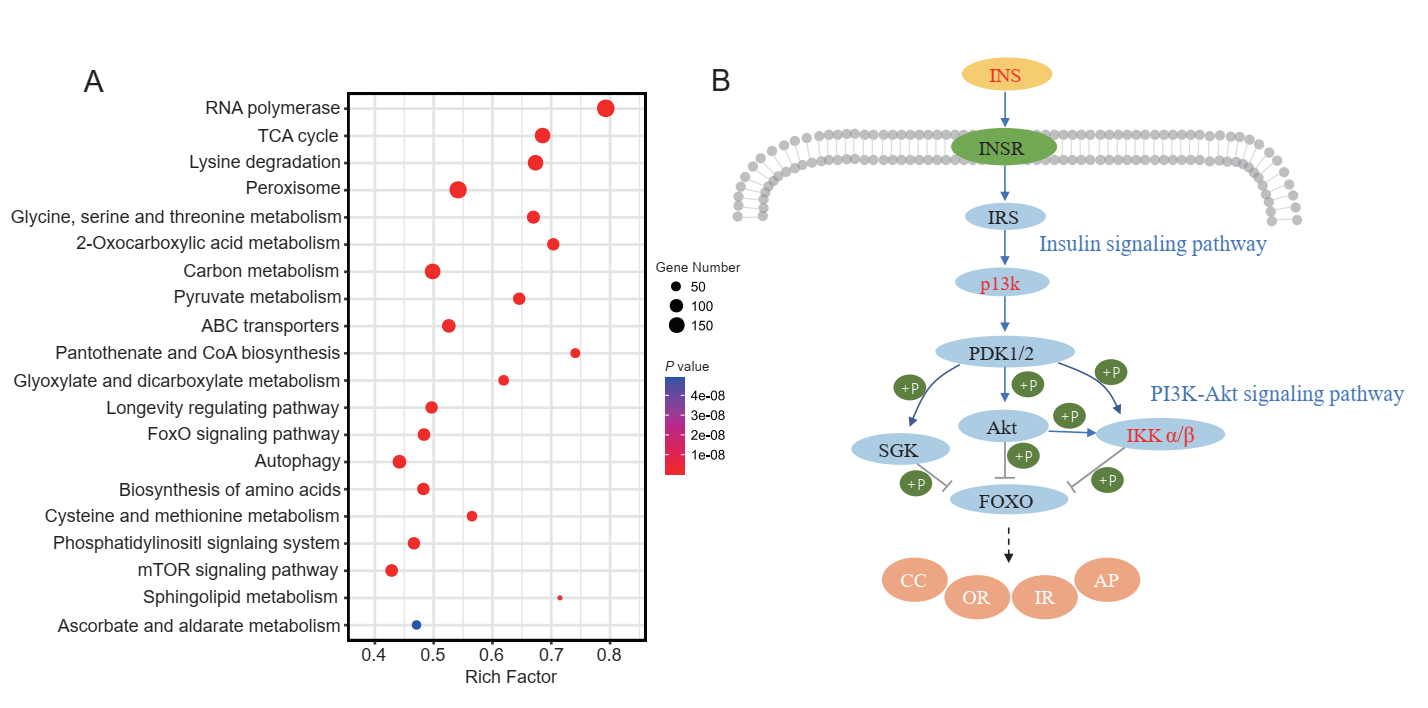


**Supplementary Figure S6. KEGG analysis of expanded gene families in *Agrostis ipsilon***. (A) Expanded gene families and their rich factor (ratio of number of differently expressed genes in the pathway to the number of all annotated genes in this pathway. Dot colors of dot represent value of *P*. (B) Key processes and related genes expanded in the forkhead box O (FoXO) signaling pathway. Expanded genes are in red. INS, insulin; INSR, insulin receptor; IRS1, insulin receptor substrate 1; p13K, phosphatidylinositol 3-kinase; PDPK1, 3-phosphoinositide dependent protein kinase-1; SGK1, serum/glucocorticoid-regulated kinase 1; AKT, RAC serine/threonine-protein kinase; IKKα/β, inhibitor of nuclear factor kappa-B kinase. CC, cell cycle; OR, oxidative stress resistance/DNA repair; IR, immune regulation; AP, apoptosis.


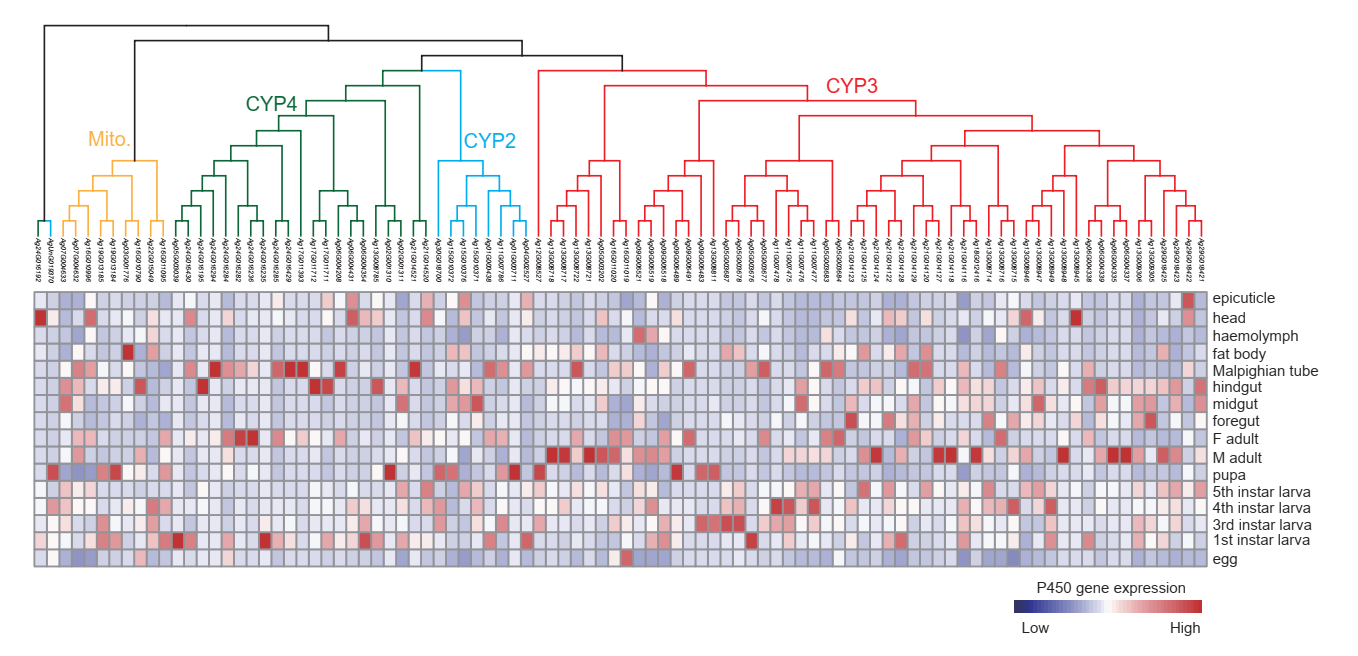


**Supplementary Figure S7. Expression profiles of P450 genes in different tissues and developmental stages of *Agrostis ipsilon*.**


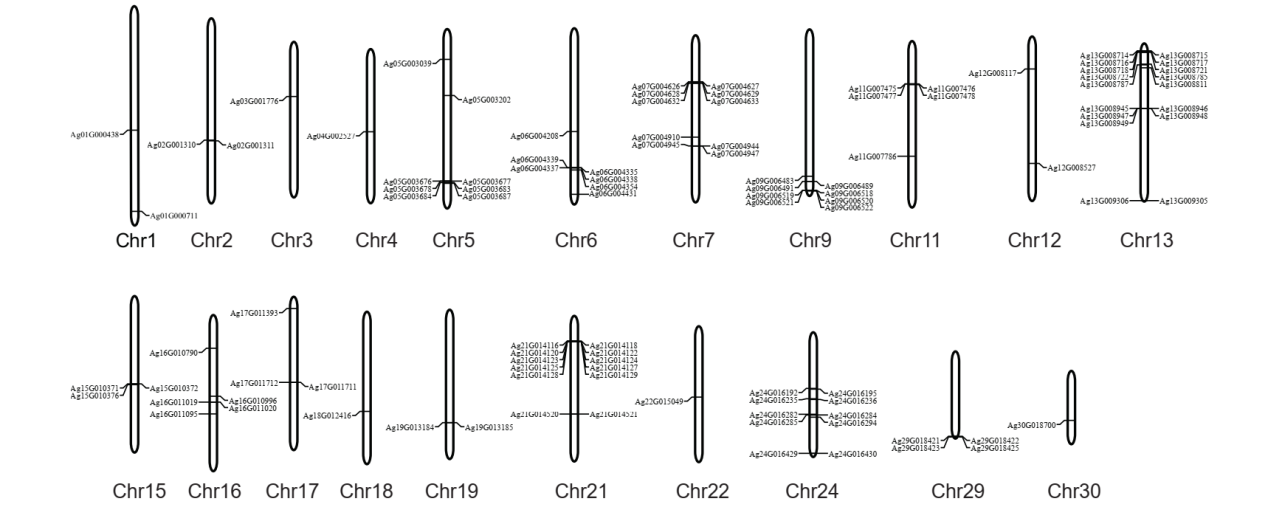


**Supplementary Figure S8. Distribution of P450 genes in the chromosomes of *A. ipsilon*.**


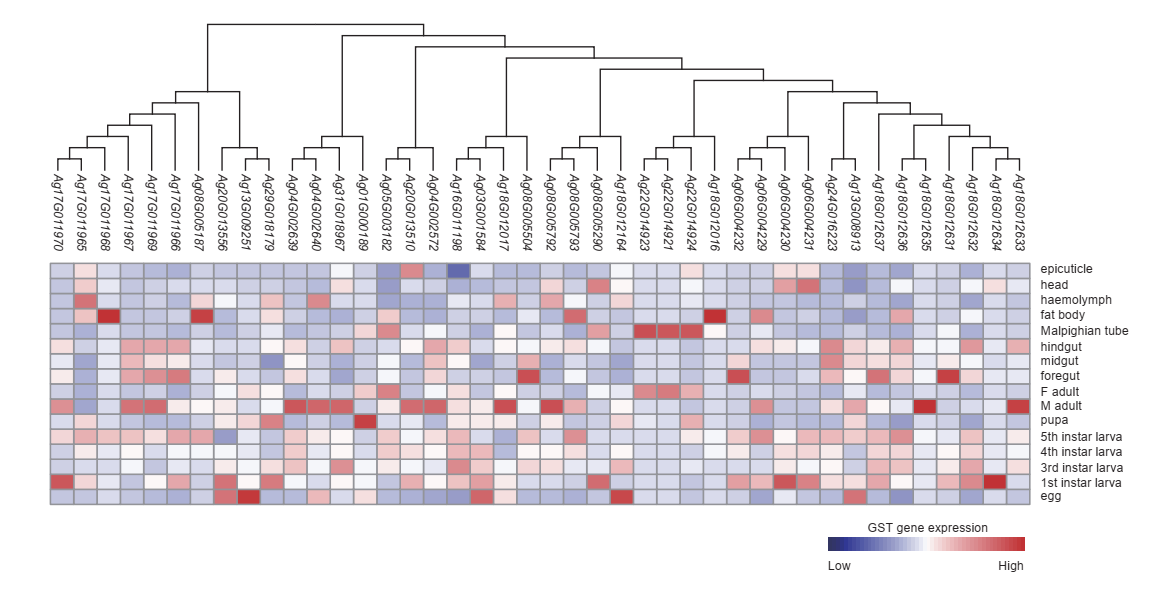
**Supplementary Figure 9. Expression profiles of GST genes in different tissues and developmental stages of *Agrostis ipsilon*.**


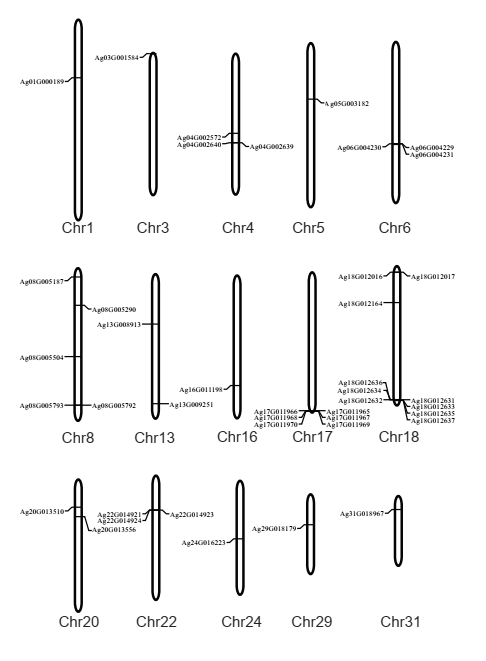


**Supplementary Figure S10. Distribution of GST genes on the chromosomes of *Agrostis ipsilon*.**


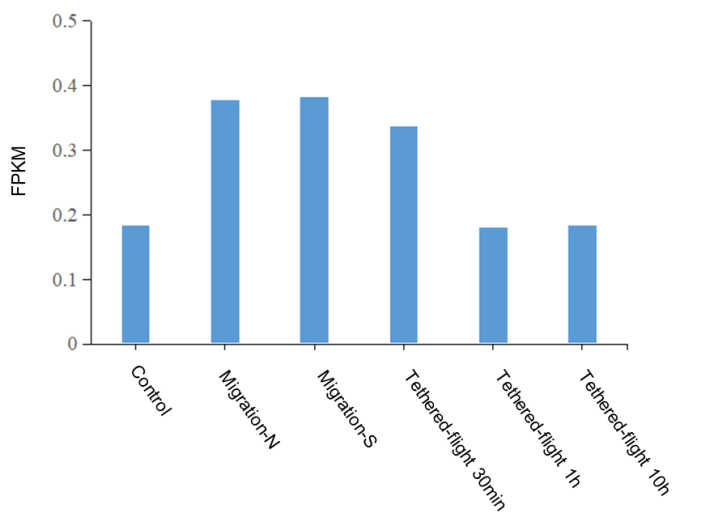


**Supplemental Figure S11. The expression level of pigment-dispersing factor (PDF) in head of migrating moths and tethered-flight moths.**


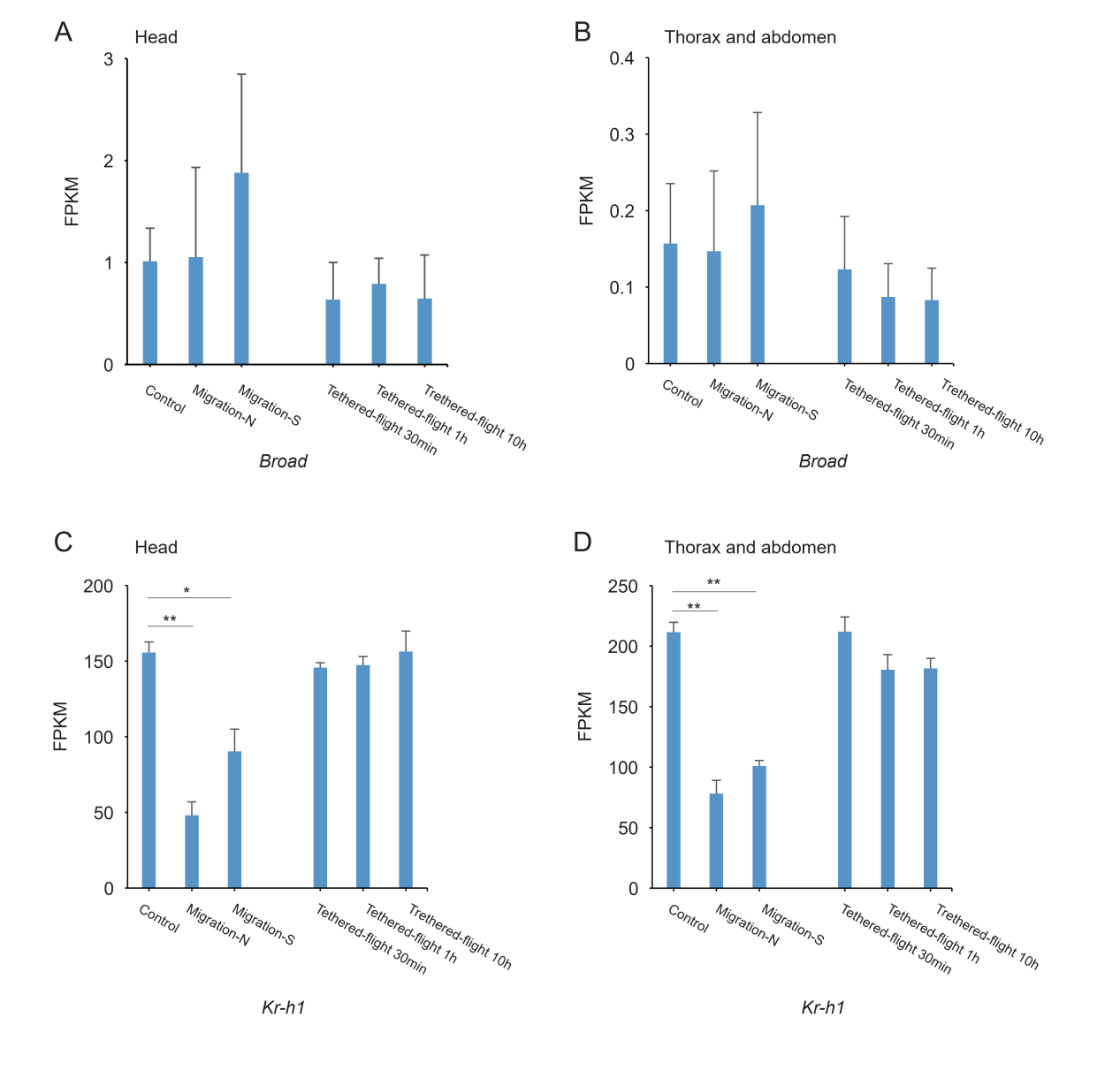


**Supplemental Figure S12. The expression level of JH signaling pathway genes, *Broad* and *Kr-h1*, in different tissues of migrating moths and tethered-flight moths (**p* < 0.05, ***p* < 0.01).**


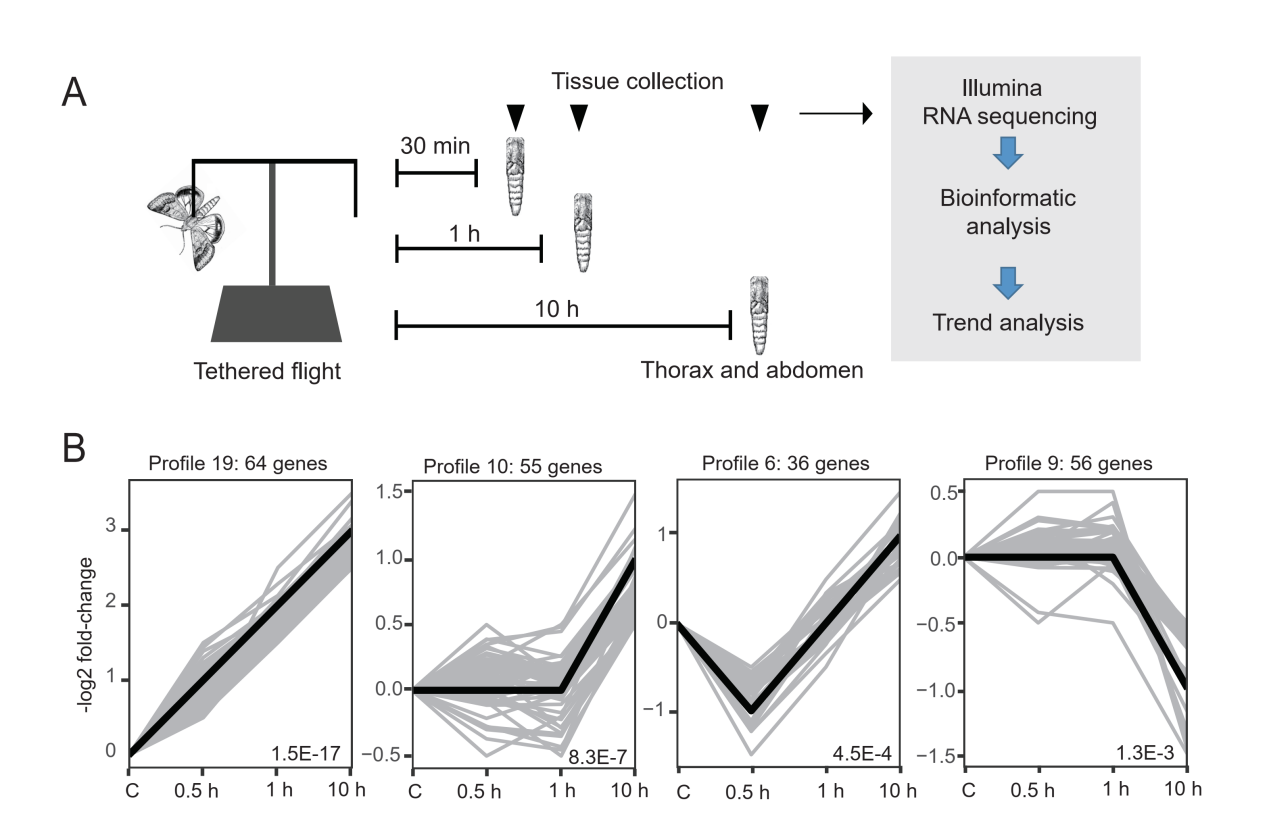


Supplemental Figure S13. RNA-seq of tethered-flight. (A) Diagram of tethered-flight experiment. (B) Significant enrichment analysis of four profiles in moths after different durations of tethered flight.


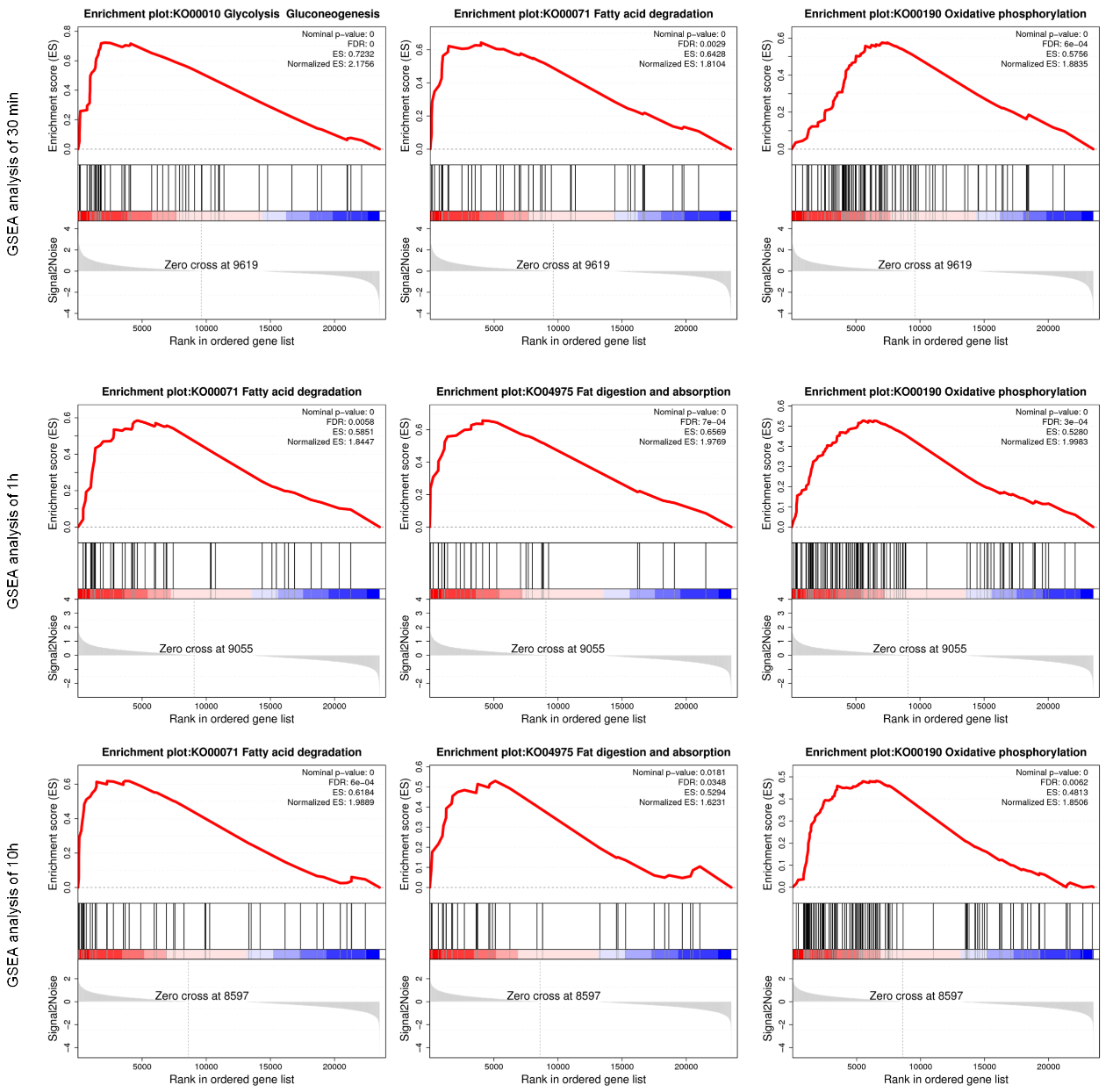


**Supplemental Figure S14. GSEA after different durations of tethered-flight moths of *Agrostis ipsilon*.** The first row represent the significant enriched pathways after 30 min of flight, including glycolysis gluconeogenesis, fatty acid degradation, and oxidative phosphorylation (FDR< 0.05). The second row represent the significant enriched pathways after 1 h of flight, including fatty acid degradation, fat digestion and absorption, and oxidative phosphorylation (FDR< 0.05). The third row represent the significant enriched pathways after 10 h of flight, including fatty acid degradation, fat digestion and absorption, and oxidative phosphorylation (FDR< 0.05).


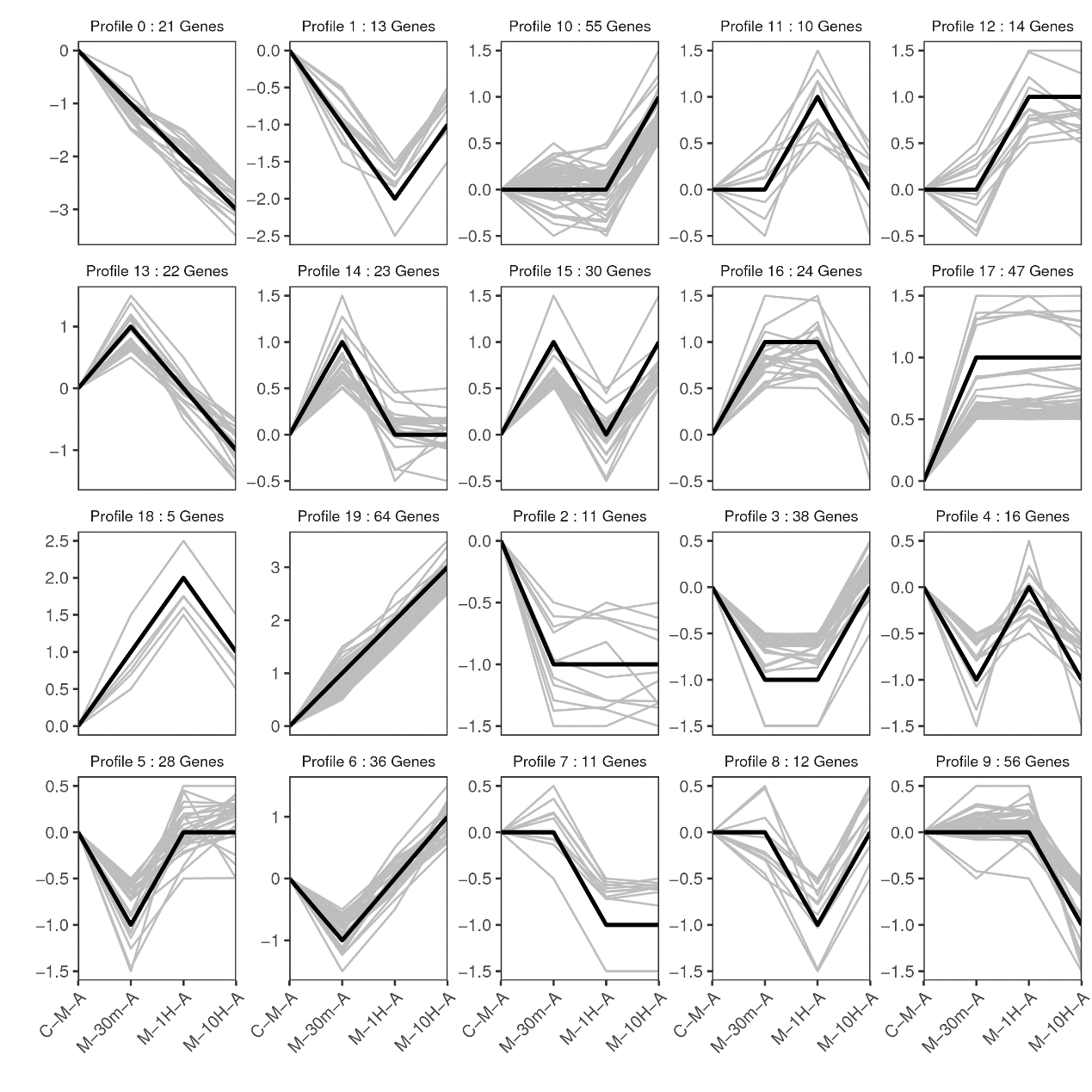


**Supplementary Figure S15. Trend analysis of DEGs after different durations of tethered flight.**


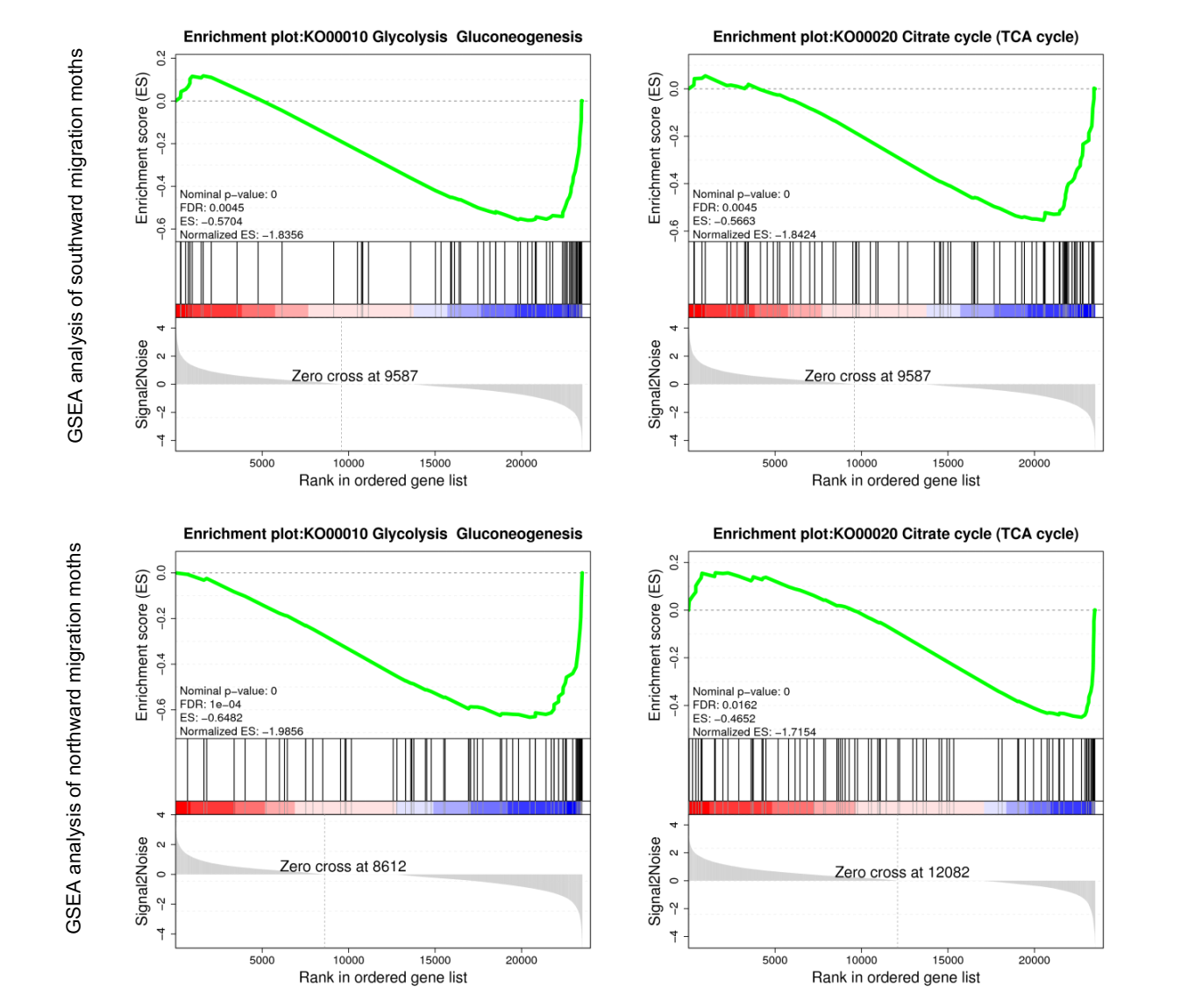


**Supplemental Figure S16. GSEA analysis of southward and northward migrating moths.**
