# Supplementary figures and images for: Chromosome-level genome of black cutworm provides novel insights into polyphagy and seasonal migration in insects
Source: BMC Biol. 2023 Jan 5;21:2. doi: 10.1186/s12915-022-01504-y (PMC9814246; doi:10.1186/s12915-022-01504-y)

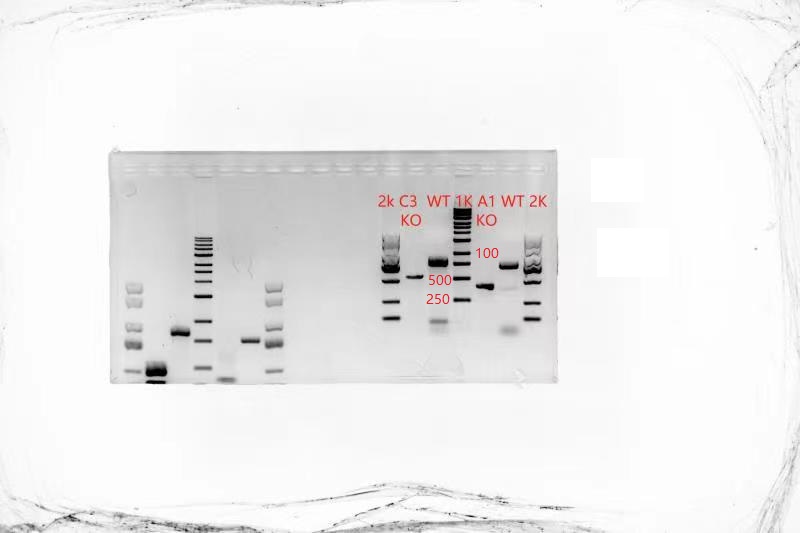

Supplement: Supplementary file 3 — Additional file 3. Raw gel image is shown in Additional File 3. [file 12915_2022_1504_MOESM3_ESM.jpg]
